# New Insights into the Therapeutic Applications of CRISPR/Cas9 Genome Editing in Breast Cancer

**DOI:** 10.3390/genes12050723

**Published:** 2021-05-12

**Authors:** Munazza Ahmed, Grace Hope Daoud, Asmaa Mohamed, Rania Harati

**Affiliations:** Department of Pharmacy Practice and Pharmacotherapeutics, College of Pharmacy, University of Sharjah, Sharjah P.O. Box 27272, United Arab Emirates; U16100699@sharjah.ac.ae (M.A.); U16103217@sharjah.ac.ae (G.H.D.); U16103316@sharjah.ac.ae (A.M.)

**Keywords:** breast cancer, CRISPR, Cas9, tumorigenesis, metastasis

## Abstract

Breast cancer is one of the most prevalent forms of cancer globally and is among the leading causes of death in women. Its heterogenic nature is a result of the involvement of numerous aberrant genes that contribute to the multi-step pathway of tumorigenesis. Despite the fact that several disease-causing mutations have been identified, therapy is often aimed at alleviating symptoms rather than rectifying the mutation in the DNA sequence. The Clustered Regularly Interspaced Short Palindromic Repeats (CRISPR)/Cas9 is a groundbreaking tool that is being utilized for the identification and validation of genomic targets bearing tumorigenic potential. CRISPR/Cas9 supersedes its gene-editing predecessors through its unparalleled simplicity, efficiency and affordability. In this review, we provide an overview of the CRISPR/Cas9 mechanism and discuss genes that were edited using this system for the treatment of breast cancer. In addition, we shed light on the delivery methods—both viral and non-viral—that may be used to deliver the system and the barriers associated with each. Overall, the present review provides new insights into the potential therapeutic applications of CRISPR/Cas9 for the advancement of breast cancer treatment.

## 1. Introduction

Breast cancer is one of the most prevalent forms of cancer globally [1]. In 2018, 2.1 million women were diagnosed with breast cancer in 185 countries, accounting for 25% of new cases [2], a number that is estimated to increase to approximately 3.2 million by 2050 [3]. Moreover, almost 630,000 deaths are attributed to breast cancer, which is approximately 6.6% of all cancer-related deaths worldwide [2]. 

Breast cancer is a malignancy where uncontrolled diving cells, most commonly arising from the epithelial cells of the mammary duct, invade the surrounding tissues and adhere to the fascia of the chest wall [4]. It can generally be classified into in situ and invasive carcinoma based on histology. Invasive breast cancer indicates the malignant proliferation of cells that have penetrated out of either the duct or lobule from which they arise, and have invaded surrounding breast tissue [5], whereas in situ refers to cancer cells confined within the site of origin [6].

**Figure 1:**
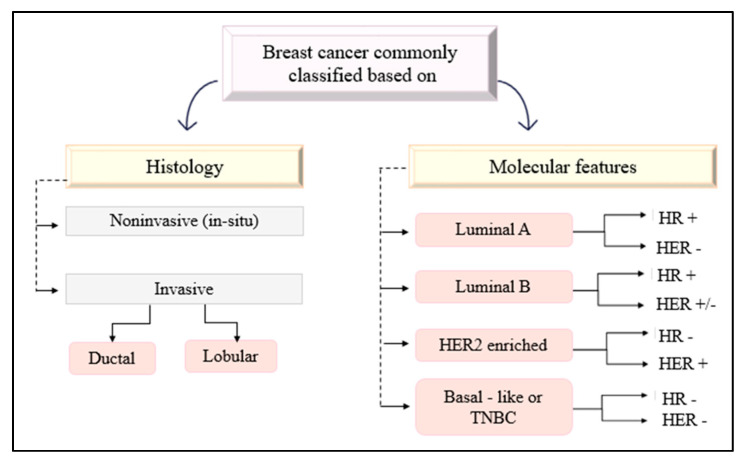
Classification of breast cancer based on histology and molecular features.

Expression of certain biomarkers such as Hormone Receptors (HR) which includes estrogen receptor (ER), progesterone receptor (PR) and Human Epithelial growth factor Receptor 2 (HER2) can further classify breast cancer into molecular subtypes (Figure 1). These include Luminal A (ER+ and/or PR+, HER2-, low Ki-67), Luminal B (ER+ and/or PR+, HER2+/−, high Ki-67), HER2 Enriched (ER-, PR-, HER2+) and Basal-like or Triple Negative (TNBC) (ER-, PR-, HER2-) [5].

Hormone-receptor and HER2 positive cancer subtypes have proven to be responsive to drugs that either have a direct effect on hormone receptors or HER2 or the pathways involved in hormonal disturbances. These drugs include tamoxifen and aromatase inhibitors and others such as trastuzumab and tyrosine kinase inhibitors [7]. TNBC patients cannot depend on such drugs, implying the need for other therapeutic strategies including surgery, radiation and/or chemotherapy [8]. Moreover, owing to its heterogeneity, breast cancer treatment requires an individualized therapeutic approach. Nonetheless, malignancies detected in early stages have a more promising prognosis, as advancements into the disease and metastasis into other organs such as the brain, bone and liver, make the cancer incurable albeit treatable. Survival outcomes at this stage are determined primarily by the site of metastasis [9], where patients with bone metastasis have shown to have the best survival rate, while brain metastasis had the least [10].

Advances in the field of genetics have led to a deeper understanding of the disease’s pathogenesis over the years, whereby identification of genetic mutations and characterization of mechanisms involved at the genomic level can lead to better targeted therapies devoid of lethal side effects. Unraveling the genetic aspects of breast cancer combined with the advancements in genetic engineering have sparked interest among researchers aiming to progress within the field. Nevertheless, limitations arising from the lack of tools to identify oncogenic drivers without activation of multiple pathways have proven to be a challenge [11]. Previous attempts in gene editing, including nuclease technologies or homing endonucleases, have now evolved into molecular techniques such as utilizing the transcription activator-like effector nucleases (TALENs) and zinc-finger nucleases (ZFNs) [12]. However, the discovery of CRISPR/Cas9 has shown notable advantages through its efficiency, practicality and versatility over the other methods [13,14].

## 2. CRISPR/Cas9 System

A major breakthrough in the field of genomics is the development of the CRISPR/Cas9 technology that has revolutionized gene editing in the 21st century. CRISPR (Clustered Regularly Interspaced Short Palindromic Repeats) was first identified in Escherichia Coli in 1987 as a group of repeated fragments comprised of 29 nucleotides that are separated by fragments of 32 nucleotides of unique varied sequence [1]. This was shown to play a role in multiple cellular processes including thermal adaptation [15], DNA repair [4] and chromosomal rearrangements [16]. In addition, a comparable 24 to 40 nucleotide short palindromic repeat sequence interspaced by a 20 to 58 varied nucleotide sequence was later identified in multiple species of bacteria and archaea, such as in Streptococcus pyogenes (S.pyogenes), Mycobacterium tuberculosis and Haloferax Mediterranean [17,18]. In 2005, researchers elucidated the homology between the short spacer fragments found on the CRISPR locus and the DNA of prokaryotic invading pathogens. Research over the years showed that CRISPR evolved with time as an adaptive immune system, protecting bacteria and archaea from foreign DNA invaders such as viruses and plasmids [19].

The CRISPR/Cas systems are grouped into 2 classes, 6 types, and 33 subtypes indicated by the involvement of the different Cas proteins within the CRISPR framework that either target DNA, RNA, or both [8,20]. The classification is summarized in Table 1 [21].

In 2013, scientists proposed the development of a targeted genome editing tool using the CRISPR/Cas9 technology found in S. pyogenes [10]. Specifically, the class 2 type II subgroup found in this species is most extensively employed for genome editing due to its simplicity necessitating merely a single Cas protein, the endonuclease protein Cas9, along with 2 RNA components, CRISPR RNA (crRNA) and trans-activating CRISPR RNA (tracrRNA). The CRISPR/Cas9 system in S. pyogenes (SpCas9) was further simplified to constitute two components, the Cas9 protein and a single guide RNA (sgRNA) through the hybridization of crRNA and tracrRNA, enabling the manipulation of the eukaryotic genome [10,22,23]. Correspondingly, the immunity provided by the CRISPR/Cas9 system can be characterized into three phases: (1) integration of phage or plasmid DNA into the CRISPR array, (2) CRISPR locus transcription to form pre-crRNA and maturation into crRNA and formation of tracrRNA, and (3) DNA manipulation [24].

The initial phase of CRISPR/Cas9 activity is the integration of short sequences of phage or plasmid DNA, termed protospacer, into the host genome which serves as a cellular memory of past infections. This enables prokaryotes to distinguish subsequent infections by these invaders as foreign, leading to silencing of the alien DNA. The acquired foreign DNA constitutes the varied region, called spacer, found on the CRISPR loci [25]. CRISPR spacer acquisition is mediated by two core proteins, Cas1 and Cas2, which are the only proteins virtually found in almost all the identified CRISPR/Cas systems [26]. A stable complex is formed between these two proteins to initiate the adaptation process; Cas1 possesses endonuclease activity that is necessary for spacer integration while Cas2 seems to play out a non-enzymatic role [27]. It has also been suggested that Cas9 plays a direct role in protospacer acquisition by recruiting Cas1 and Cas2 to potential targets [28].

**Figure 2:**
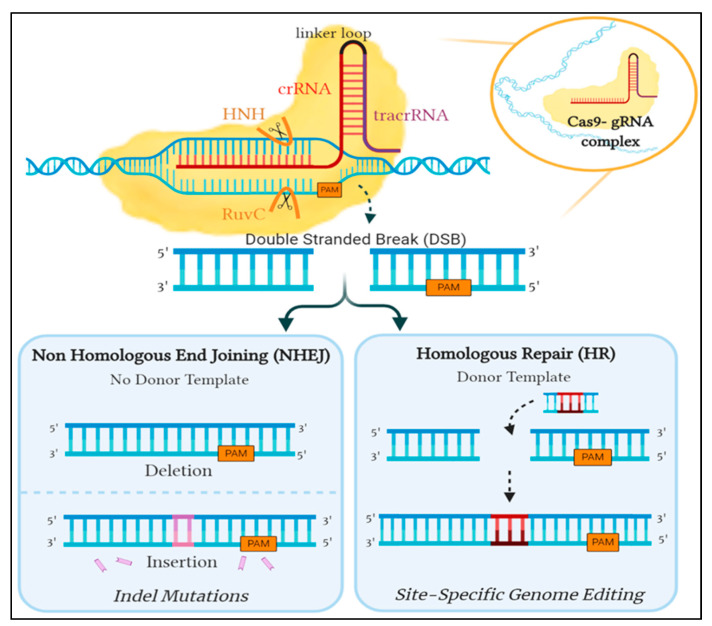
An overview of the repair mechanism associated with induced Cas9 double-stranded DNA break. Cleavage is induced by the binding of Cas9- gRNA complex to its complementary sequence on foreign DNA. In eukaryotes, this is amended by either of two mechanisms: the error-prone Non-Homologous End Joining (NHEJ) or Homologous Repair (HR), which is utilized for genome editing by providing a donor template. *Created with Biorender.*

The second phase begins with the transcription of the CRISPR locus to generate pre-crRNA, a long RNA molecule that contains sequences complementary to those of the spacers and repeats. The tracrRNA is the second RNA molecule needed and is essential for pre-crRNA maturation, it is transcribed from a genomic locus located upstream of the CRISPR locus [29]. The tracrRNA contains a segment that is homologous to the cognate sequence of the CRISPR locus; therefore, is able to bind to the 3′ end of the pre-crRNA forming a double-stranded RNA molecule [30]. Subsequently, the pre-crRNA:tracrRNA double-stranded RNA is cleaved by recruited cellular ribonuclease III (RNase III) responsible for the recognition and cleavage of double-stranded RNA molecules [29]. A second cleavage takes place whereby the 5′ end of the RNA sequence is cut, yielding a mature crRNA:tracrRNA (gRNA) complex ready to associate with a Cas protein, with each individual crRNA fragment containing a unique spacer sequence that is around 20 nucleotides in length [30,31]. The resulting gRNA complex binds to the Cas9 protein, creating a Cas9:gRNA effector complex capable of DNA interference to complete the CRISPR mediated immunity. The Cas9 protein is a dual RNA-guided endonuclease enzyme having a bi-lobed structure, the α-helical recognition (REC) lobe and the nuclease lobe, with the RNA complex situated in between. The latter is comprised of two nuclease domains, an HNH domain responsible for cleaving the complementary DNA strand to crRNA and a RuvC-like domain which cleaves the non-complementary DNA strand. On the other hand, the REC lobe contains an arginine-rich bridge that is essential for RNA interaction and joining the two lobes together [32,33,34]. Once Cas9 is activated by binding to the gRNA complex, it scavenges for any invading nucleic acid sequences that show complementarity to the crRNA. Therefore, CRISPR/Cas9 initiates a double-stranded cleavage at a specified DNA sequence site following base pairing of crRNA to the target site [35] (Figure 2).

However, the prospective target sequence is only valid if a short sequence known as Protospacer Adjacent Motif (PAM) is present directly after the binding location of crRNA. The presence of PAM is the underlying factor that determines preference between self and non-self DNA. Although the CRISPR array contains spacers that are identical to foreign DNA, the CRISPR genome is not affected by its own mechanism as the spacers do not lie immediately next to a PAM sequence [36]. In addition, the PAM sequence identified by Cas9 varies between microorganisms; SpCas9 specifically recognizes 5′-NGG-3′ [35], resulting in a blunt-end double-strand break occurring upstream by three base pairs in the PAM sequence [30]. The guanine dinucleotide [32] of PAM found on the non-complementary strand aids in its recognition by interacting with two crucial arginine residues. Further interactions form a bend in the target DNA assisting in the unwinding of the helical structure which propagates cutting of the intruder DNA [37]. This disruption in the invading pathogen is deemed to be detrimental to its existence and is ultimately what provides protection for the prokaryote. 

This guided interference into the DNA sequence inspired researchers to exploit the system with hopes of achieving precise genome editing. Unfortunately, the CRISPR/Cas9 system in prokaryotes utilizes components not inherently present in eukaryotes, prompting the need to optimize the S. pyogenes’ CRISPR/Cas9 system. The modifiable crRNA is merged with the tracrRNa to form sgRNA, which works similarly to the gRNA complex as it guides Cas9 to the target sequence site and triggers the cleavage of both DNA strands. The double-strand DNA breaks induced by Cas9 can then be amended by one of two DNA repair pathways: either the non-homologous end joining (NHEJ) or the homology-directed repair (HDR) [38]. NHEJ ligates the broken ends together, however, this pathway is error-prone and could lead to insertion/deletion (indel) mutations resulting in an ineffective gene. In contrast, HDR uses a neighboring homologous sequence as a template to mend the break. This method can be exploited to potentially introduce targeted edits at a precise location into the DNA sequence by providing a donor template attached to sgRNA for repair [30] (Figure 2). 

As the machinery behind the whole CRISPR/Cas9 system relies on the complementarity between crRNA and the target sequence, in addition to the presence of PAM [35], specificity is crucial when generating a sgRNA. In case the desired target is inaccurately outlined, Cas9 can bind and cause an off-target cleave of the sequence, leading to unintended mutations that could be consequential. However, as long as the target sequence is identified, several CRISPR software tools are available to facilitate the design of an optimal sgRNA to achieve precise cleavage with minimal off-target effects [39]. Owing to the versatility in sequencing sgRNAs, attachment of the Cas9:gRNA complex to various sites is plausible. This merits the CRISPR/Cas9 system to reconstruct a multitude of loci concurrently [40]. 

## 3. Potential CRISPR/Cas9 Targets

### 3.1. Genes Involved in Tumorigenesis

Mutations in the genes regulating several cellular processes, including but not limited to cell survival, proliferation, motility and apoptosis, deregulate the gene normal function which, as a result, could promote uncontrolled cellular growth, giving rise to tumor formation). Discussed below are critical genes involved in the pathogenesis of breast cancer with supporting studies employing CRISPR/Cas9.

#### 3.1.1. BRCA1

**Figure 3:**
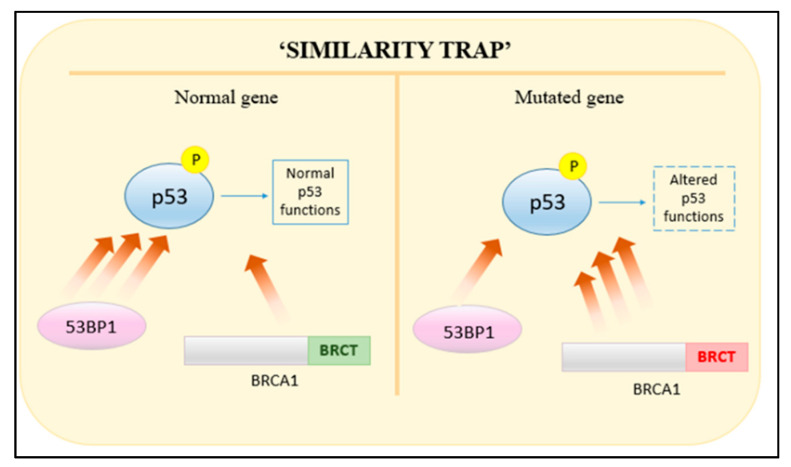
Reversal of affinities between mutated BRCA1 and 53BP1 towards phosphorylated p53.

The BRCA1 gene is expressed in a number of tissues, including ovarian and breast tissue. Acting as a tumor suppressor, it is involved in multiple cellular regulatory pathways including gene transcription regulation, ubiquitination, cell-cycle progression and DNA-damage response; the latter being a pathway in which BRCA1 plays a pivotal role [41]. Such versatility in its role is a result of possessing multiple functional domains that aid in checkpoint regulation, single-strand annealing (SSA), HR and NHEJ [42]. These include the N-terminal RING (Really Interesting New Gene) domain, the BRCT (BRCA1 C-terminal) domain and exon regions 11–13. Site I and Site II are Zn2+ binding loops responsible for stabilizing RING finger structures; containing four cysteine and three cysteine residues with one histidine residue, respectively. Mutations in the cysteine residues result in altered functions such as decreased ubiquitin ligase activity, which has been shown to increase cancer risk [43,44]. The BRCT domain in BRCA1 functions to regulate interactions of phosphoproteins with BRCA1 as well as facilitating non-phosphoprotein interactions with DNA binding. Mutations of a residue would therefore hinder the role of BRCA1. Such is the case of BRCA1 falling into the “similarity trap” (Figure 3) [45,46]. Normally, 53BP1 (p53 binding protein I) has a higher affinity to phosphorylated p53 than BRCA1. Mutated BRCT domains in BRCA1 reverse the affinity between 53BP1 and BRCA1, which then alters p53 function, possibly precipitating cancer [46].

Furthermore, several variants and non-coding regions are yet to be discovered owing to the complexity within the BRCA1 genomic structure. A study implementing the CRISPR-mediated cytosine Base Editor 3 (BE3) to induce targeted T: A conversions from C: G assessed the functionality of variants of uncertain significance (VUSs) and identified loss-of-function (LOF) variants through high-throughput screens; which can aid in the reclassification of BRCA1 (VUSs). The HAP1 cell line was used to introduce LOF mutations, however prior to that, to demonstrate the feasibility of the experiment, transfection of BRCA1-targeting gRNAs into HAP1-Cas9 cell lines was carried out to derange BRCA1. 

Since changes in cell viability due to loss-of-function (LOF) mutations in BRCA1 variants can be used to assess the function of BRCA1, targeted deep sequencing was used to measure mutation frequencies, where the relative indel frequencies decreased substantially with time. BE3-expressing HAP1 (HAP1-BE3) and Cas9-expressing HAP1 (HAP1-Cas9) cell lines were generated with lentiviral particles. After inducing nucleotide substitution mutations by HAP1-BE3 cell lines, a collective gRNA targeting BRCA1 library was designed to accomplish CRISPR-based high-throughput screens [47].

#### 3.1.2. BRCA2

Multiple studies have demonstrated an overlap between cancer outcomes and BRCA1/BRCA2 carriers [48] despite the lack of homology between these genes [49]. The BRCA2 domains are essentially associated with RAD51; a protein related to homologous repair (HR) [50]. DNA repair mechanisms are maintained by BRCA2 through its cyclin-dependent kinase (CDK) interaction with RAD51 to aid in HR—an established role of BRCA2 [51]. In addition, BRCA2 protects nascent strands during replication from degradation [52]. 

Paradoxically, however, the transmission of replication stress responsible for precancerous lesions to the succeeding cell cycle due to BRCA2 deficiency causes missegregation, as 53GP1 nuclear bodies are formed at the G1 phase. This eventually causes cell inviability as the p53-dependent G1 is seized, as was demonstrated by Feng W. and Jasin M. through CRISPR/Cas9-mediated gene targeting of BRCA2 [53]. These results point out a potential barrier that must be tamed for the commencement of tumorigenesis.

#### 3.1.3. HER2

The Human Epidermal Growth Factor 2 (HER2 or HER2/neu or ERBB2) gene is an oncogene that encodes for the HER2 protein, a membrane receptor tyrosine kinase that is one member of four in the ERBB family (HER1-4) found on breast cells. Amplification of HER2 and the resulting overexpression of its protein is found to be important in the carcinogenesis of the HER2-positive breast cancer subtype [54]. The HER2 signaling pathway, among others, is a major driver of tumor cell proliferation and survival in this breast cancer subtype, and an effective therapeutic target of the monoclonal antibody trastuzumab [55,56]. The HER2 protein, along with the other ERBB family members, consists of three domains: an extracellular, transmembrane and intracellular domain. Once a ligand binds to the extracellular domain, the HER protein will dimerize and result in autophosphorylation of the intracellular domain, which in turn interacts with other signaling molecules that initiate the activation of a variety of downstream signaling pathways involved in cell proliferation, survival and opposes apoptosis. Interestingly, the HER2 protein does not have an associated ligand but rather relies on heterodimerization with any of the other three HER proteins or homodimerization when overexpressed for activation [57]. In addition, heterodimers with the HER2 protein possess the greatest signaling activity, as it has the strongest kinase activity [58]. 

Targeting of HER2 via CRISPR/Cas9 led to inhibition of cell proliferation and carcinogenesis of breast cancer cells. Exons 5, 10 and 12 of HER 2 were specifically targeted by selective gRNAs, these exons exist in all HER2 isoforms and are responsible for encoding parts of the extracellular domain. Cas9, along with three gRNA, was introduced into HER2+ breast cancer cell lines BT-474 and SKBR-3 and the HER2- breast cancer cell line MCF-7. The co-expression of Cas9 and gRNAs substantially suppressed cell growth in HER2+ cell lines but not in HER2- cell lines, a result that is comparable to that obtained from trastuzumab treatment. In addition, the introduction of Cas9 and gRNAs to a soft agar colony formation assay caused a considerable reduction in colony formation. These results indicate that utilizing CRISPR/Cas9 to target HER2 yields decreased cell proliferation and carcinogenesis, though the effect is limited to HER2+ cell lines [55]. 

#### 3.1.4. TP53

A widely acknowledged tumor suppressor is the p53 protein, encoded by the TP53 gene. Regrettably, mutations in this gene account for half of the cancer cases, making this the most commonly mutated gene in cancer [59]. In many instances, an amino acid substitution in the p53 protein ensues from one nucleotide mutation in the TP53 gene. These are usually frameshift mutations with the resulting protein possessing non-functional tumor-suppressing and diminished transcriptional activity [60]. Additionally, the mutation stimulates oncogenesis and can affect advanced cancer stages and cancers unresponsive to treatments [61]. Some malignancies, despite possessing the wild-type (WT) TP53 gene, generate multiple cancers with inactive p53 proteins. Post-translational modifications through overexpression of epigenetic regulators in this protein damage its tumor-suppressive properties. Reactivating p53 by inhibiting the contributing epigenetic factors such as methyltransferases and Aurora A kinases could prove to be beneficial towards cancer suppression [62]. 

However, studying the effect of inducing Cas9 expression in cells, Enache et al. observed p53 pathway activation in Cas9-expressing cell lines. When WT TP53 expressing and TP53 LOF mutated cell lines were compared, the former cell line was most commonly found to have the pathway activated, undeniably because the well-established role of p53 in TP53 LOF cell lines is inactivated. However, not only was there an increase in the number of cells with DNA DSBs upon Cas9 expression but, in addition to other genes, TP53 mutations were observed in Cas9 expressing cell lines. The study also indicated that upon Cas9 expression, cells harnessing the TP53 LOF mutation paradoxically showed increased cell growth; an effect exclusive to TP53 and not in other tumor-suppressing genes, indicating a possible limitation when conducting experiments on TP53 with Cas9 [63]. In this context, it is important to note that findings on targeting TP53 with CRISPR/Cas9 could be extended to other types of cancers knowing that TP53 is a commonly mutated gene in various types of cancer.

#### 3.1.5. TP53PB1

Since the concept of defective DSB repair by the LOF of BRCA1 protein aids in chemotherapy of BRCA1/2 deficient cancers by hyper-sensitizing cells to Poly (ADP-ribose) polymerase (PARP) inhibitors, a study [64] set out to understand the mechanisms by which resistant clones of PARP inhibitors arise, specifically focusing on the loss of 53BP1-encoded by the TP53BP1 gene and its associated co-factors. It is important to note that the chief binding partner of BRCA2, the PALB2 (partner and localizer of BRCA2) gene colocalizes with BRCA2 and aids in its stabilization [65].

It encodes for the PALB2 proteins which bind to BRCA1 and BRCA2 proteins, thereby facilitating the repair of DSBs through homologous recombination [66,67]. Specifically, PALB2 links the BRCA complex (BRCA1-PALB2-BRCA2-RAD51), hence aiding the role of RAD51 in the strand invasion step of homologous recombination [68]. This breast cancer susceptibility gene, therefore, is vital to maintaining the integrity of the genome. Lack or dysfunction thereof would predispose to breast cancer [69]. Nonetheless, the role of 53BP1 in the multiple HR stages was characterized in depleted cells of BRCA1, PALB2 or BRCA2 by short-interfering RNA (siRNA) technology, where HR repair was shown to be strictly inhibited. 

However, the depletion of 53BP1 did not restore HR in PALB2 or BRCA2 deficient cells. On the contrary, HR was enhanced in BRCA1/53BP1 depleted cells. To determine whether such disproportional preferences in HR repair could be on account of partial 53BP1 depletion, CRISPR/Cas9 mediated knockout (KO) of the 53BP1 gene was performed, followed by HR assays. PALB2 depletion almost entirely abolished HR both in 53BP1-sufficient and 53BP1-KO cells, indicating that 53PB1 loss reinstates HR in BRCA1 deficient cells, but not in PALB2 deficiency [64]. 

The aforementioned partial nature of HR depicts that in BRCA1 deficiency, 53PB1 is not proficiently displaced from the chromatin nearby the DSBs [70]. It is still bound to nucleosomes and acknowledged by multiple chromatin-interacting proteins. Loss of 53PB1, on the other hand, exposes its now-vacant nucleosome surfaces for interaction with scarce or even factors avidly binding to the surfaces, such as PALB2. 

Briefly, the lack of 53BP1 in BRCA1 deficiency creates a preferable setting for the recruitment of PALB2. Therefore, it was shown that deficient cells of BRCA1/53BP1 led to the accumulation of PALB2 into the damaged DNA foci. Such findings elucidate the role of DNA repair by PALB2 in clinically significant BRCA1/53BP1 deficient cells [64].

#### 3.1.6. MKI67

Ki-67 is a widely used oncogenic biomarker as it is exclusively expressed in proliferating the cells of vertebrates [71]. This nuclear protein, encoded by MKI67, is utilized to grade tumors in histopathology and can be used as a prognostic marker [72]. However, recent studies have invalidated the claims of Ki-67 playing a role in cancer proliferation [68,73,74,75]. Since the proliferation of Ki-67 is controlled by regulators of the cell cycle such as CDKs and B-Myb [74,76,77], Ki-67 in itself is not overexpressed in cancers. Nevertheless, Ki-67 might prove to be essential in carcinogenesis by other means. Its ability to organize heterochromatin is evident by the disruption of nucleoli and centromeres in Ki-67 KOs and the formation of heterochromatin ectopically in its overexpression [74]. 

Widespread changes in the transcriptome were observed upon the knockout of Ki-67, which indicated that, instead of Ki-67 directly governing specific transcription factors, it interacts with multiple chromatin regulators to give such ‘global transcriptome changes’. This was tested on cell lines simulating TNBC [78,79]. CRISPR/Cas9 mediated gene KO of MKI67 showed no change in the proliferation rates of the tested cell line but rather, the KO caused a genome-wide alteration in the gene expression. In addition, being an intrinsically disordered protein (IDP), the study also concluded that the expression of Ki-67 has an impact on contributing pathways involved in carcinogenesis, ranging from initiation and progression to metastasis. Consequently, the lack of enzymatic activities within the IDP will prove to be an obstacle in the context of finding therapeutic targets. However, inhibiting its effectors and interacting proteins could deem to be of therapeutic value [79]. Similar to TP53, as MKI67 is a proliferation maker in various malignancies, findings on targeting MKI67 by CRISPR/Cas9 can have broader applications that encompass several types of cancers.

### 3.2. Genes Involved in Metastasis

A major life-threatening problem of cancer cells, particularly those of breast cancer, is their tendency to metastasize to remote tissues across the body, making them responsible for the majority of cancer-related mortalities [80]. The metastatic process begins with the tumor cells departing from their site of origin, followed by intravasation into the bloodstream through the extracellular matrix (ECM) [81,82] where the circulating tumor cells (CTC) work to overcome several cellular obstacles including defensive immune cells. It then attaches to a secondary site, where it has to acclimate for its survival and further develop into a secondary tumor [83,84]. Epithelial-to-Mesenchymal Transition (EMT) is suggested to aid the metastatic process by decreasing the polarity of epithelial cells, yielding an aggressive mesenchymal cell that has the ability to migrate and invade with decreased susceptibility to apoptosis [85,86,87]. 

#### 3.2.1. MIEN1

Migration and invasion enhancer 1 (MIEN1), until recently named C35 or C17orf37, is a novel breast cancer oncogene that is extensively expressed in all types of breast cancers primarily in HER-2 and luminal B subtypes [88]. MIEN1 is present specifically in the ERBB2 amplicon and is fundamental in regulating the migration and invasion of cancer cells [89]. MIEN1 is involved in several pro-metastatic signaling processes including gene expression through Protein kinase B (Akt) activation [90]. Physiologically, MIEN1 is associated with the formation of filopodia to accelerate cellular motility during tumor dissemination, in addition to controlling cell apoptosis. Despite this, the exact part MIEN1 plays in tumorigenesis and metastasis remains ambiguous. In its C-terminal, MIEN1 constitutes a CAAX motif that acts as a substrate for prenylation, which is the process of covalently adding hydrophobic isoprenoid groups post-translationally to proteins, enhancing their hydrophobicity and accelerating the migration of cells. MIEN1 is prenylated by the enzyme Geranylgeranyltransferase-I (GGTase-I), subsequently promoting protein–inner membrane interactions along with directional migration [91]. The CRISPR/Cas9 tool was employed for precise targeting in MIEN1 gene deletion of MDA-MB-231 breast cancer cell lines where a segment of the gene was knocked using a two sgRNA co-transfection approach to ensure stability. Based on the results, this co-transfection leads to elevated on-target efficiency whereby most of the cell lines demonstrated no expression of the MIEN1 protein. Moreover, the results also inferred no apparent discrepancies in tumor proliferation, morphology or vitality between the MIEN1 KO cells and the original cell lines. The study illustrated the significance of further research into the specifics of MIEN1-mediated oncogenesis in order to substantiate its prospects as a clinical target and biosignature in breast cancer and how CRISPR could be integral in this process [92].

#### 3.2.2. CX3CR1

CX3 chemokine receptor 1 (CX3CR1) is a protein encoded by the CX3CR1 gene and has been implicated in the dissemination of cancer cells and facilitation of cell survival and viability [93]. The only chemokine ligand of CX3CR1 is Fractalkine (FKN or CX3CL1), found expressed as either a transmembrane protein or a soluble protein, where it functions as a chemoattractant [94]. Therefore, the interaction between FKN and CX3CR-expressing CTC is crucial to the precipitation of secondary cancer lesion formation in the skeleton and other soft-tissue organs [93]. Examination of breast cancer specimens of luminal A, luminal B, HER2 and TNBC subtypes all indicated comparable expression of CX3CR1 [95]. Breast cancer patients with bone metastasis show no correlation to breast cancer subtypes, which implies the large role of CX3CR1 in skeletal dissemination [96].

A CRISPR-mediated silencing of CX3CR1 transcription in MDA-MB-231 cell lines led to the ablation of in vitro CX3CR1 protein expression. Upon the introduction of these cells in mice, there was a significant reduction in skeletal and lung metastasis [93]. Comparable results were obtained from FKN knockout transgenic mice (also grafted with MDA-MB-231) indicating that CX3CR1 is directly involved in the lodging of breast cancer cells to the bone [97,98]. Therefore, interfering with the pairing of CX3CR1 with FKN, through the deletion of CX3CR1, can drastically limit the capability of breast cancer circulating tumor cells to disseminate and cause secondary tumors [93], particularly in the bones.

#### 3.2.3. CXCR2

Interleukin 8 (IL-8 or CXCL8) is a chemoattractant cytokine secreted from various cells such as leukocytes, endothelial cells, fibroblasts and malignant tumor cells under certain environmental stressors and takes part in malignant cell migration, proliferation and angiogenesis [99,100]. IL-8 produces an effect upon binding to its receptors, CXC chemokine receptors 1 and 2 (CXCR1 and CXCR2), which are heterodimeric receptors primarily expressed on immune cells such as neutrophils, but may also be present on the surface of various tumor cells [101]. Binding with CXCR2 (encoded by the CXCR2 gene) facilitates cell migration [102] and is therefore reported to play a vital role as a receptor for tumor metastasis [103]. 

A significant increase in proliferation and migration was observed in MDA-MB-231 cell lines in conditioned media of fibroblasts and macrophages treated by tumor-conditioned media (TCM) of TNBC. This suggests that the migration and proliferation of TNBC cells is enhanced by the crosstalk among TNBC cells and either fibroblasts or macrophages. Several factors were secreted in conditioned media of fibroblasts and conditioned media of macrophages that were treated with tumor-conditioned media of TNBC. However, the secretion and expression of IL-8 was highly upregulated by both fibroblasts and macrophages, suggesting it could be a key factor that fosters TNBC cell proliferation and migration. A CRISPR/Cas9 mediated knockout of CXCR2 in MDA-MB-231 cell lines led to a significant decrease in tumor cell proliferation and migration, compared to the wild-type TNBC cell line. A decrease in metastasis was also observed in the xenograft mouse model. Therefore, these observations indicate that the IL-8-CXCR2 axis is involved in TNBC cell growth and metastasis through crosstalk of TNBC cells, fibroblasts and macrophages [104]. 

#### 3.2.4. CXCR4 and CXCR7

CXC Motif Chemokine Ligand 12 (CXCL12), otherwise known as Stromal Cell-Derived Factor 1 (SDF1), is a chemokine protein that can regulate cell proliferation, motility and angiogenesis through its interaction with CXC Chemokine Receptor 4 (CXCR4) and CXC Chemokine Receptor 7 (CXCR7) [105]. In addition, it is also highly expressed in organs such as the bone marrow, liver and lungs, allowing simple metastasis of breast cancer cells to such organs [106]. CXCR4 is a transmembrane G-protein coupled receptor encoded by the CXCR4 gene. Attachment of CXCL12 to CXCR4 activates a plethora of downstream signaling pathways resulting in various responses such as cell proliferation, survival, and migration [107]. The CXCL12/CXCR4 axis is said to have a critical role in breast cancer metastasis [105]. 

In addition to CXCR4, CXCL12 can also bind to CXCR7, a receptor that is overexpressed within the primary tumors vascular system and holds a more elaborate role in breast cancer progression [108,109]. CXCR7 fosters tumor angiogenesis by its influence on cancer cells to secrete vascular endothelial growth factor (VEGF) and promoting metastasis by augmenting cancer cell adhesion to endothelial and fibrin cells [109,110,111]. Overexpression of CXCR7 in breast cancer tissue promotes cell proliferation, invasion and metastasis, particularly to the lungs [111]. Moreover, CXCL12 along with its receptors CXCR4 and CXCR7, are overexpressed in TNBC compared to other subtypes and are correlated to metastasis and poor prognosis [112].

The CRISPR/Cas9 system was utilized to perform single-gene knockout and co-knockout of CXCR4 and CXCR7 genes in the TNBC cell line MDA-MB-231. The single-gene knockout of either CXCR4 or CXCR7 led to no protein expression and caused a significant inhibition of TNBC growth, proliferation, invasion and migration in vitro. However, co-knockout of CXCR4 and CXCR7 caused a more substantial decrease effect as opposed to single-gene knockout, suggesting a synergistic relationship between the two receptors in TNBC progression. These results indicate that CXCL12, CXCR4 and CXCR7 all have an important role in TNBC progression; therefore, co-knockout of both receptors may be a possible therapeutic target in TNBC treatment [106].

#### 3.2.5. MAP3K11

The mitogen-activated protein kinase (MAPK) pathway is involved in the regulation of multiple cellular processes including survival, proliferation, differentiation, motility and apoptosis [113]. This signal transduction is highly regulated by a relay of three protein kinases, MAP kinase kinase kinase (MAP3K), MAP Kinase Kinase (MAP2K) and MAP kinase (MAPK). The activated MAPK proteins can phosphorylate substrates within the cytosol or regulate transcription factors through nuclear translocation. Alteration or overexpression of these MAPK proteins or their upstream regulators results in upregulation of the signal transduction pathway leading to a sustained activation signaling for cancer cells [114].

Mixed-lineage kinase (MLK3) is a MAP3K that has been shown to be overexpressed in TNBC and is critical for its metastasis [115]. In addition, MLK3 is able to activate the activator protein 1 (AP-1) transcription factor—a heterodimer comprised of JUN, FOS, Activating transcription factor (ATF) and MAF that regulates gene expression and controls numerous cellular processes. Furthermore, the FOS-related antigen 1 (FRA1) belonging to the FOS family of proteins is regulated by MLK3 and is found in high amount in TNBC cells and plays relevant roles in tumor cell proliferation and invasion [116]. In addition, FRA1 regulates matrix metalloproteinase (MMP) 1 and 9, which are zinc-dependent endopeptidases involved in the degradation of extracellular matrix protein, aiding in cancer cell invasion and metastasis by remodeling the extracellular matrix [117]. All these factors that facilitate TNBC metastasis are regulated by MLK3 signaling. CRISPR/Cas9 depletion of MLK3 in highly metastatic murine TNBC 4T1 cells led to reduction of FRA-1 and consequently MMP1 and MMP9, which resulted in impairment of tumor cell invasion and migration [118].

#### 3.2.6. TNFRSF11B

Osteoprotegerin (OPG) is a cytokine belonging to the Tumor Necrosis Factor (TNF) superfamily encoded by the TNFRSF11B gene and is broadly known for its pre-emptive role in osteoclastic regulation through the RANK Ligand (RANKL). This ligand is released by osteoblasts and normally binds to the RANK receptors found on the surface of osteoclasts. OPG interacts with RANKL thereby inhibiting the interaction between the ligand and receptor, as a result diminishing both bone reabsorption and osteoclast development [119]. Comprehensive research suggests that OPG is an important tumor modulator and studies on breast cancer cell lines have shown a 40% expression of OPG compared to normal breast cells [120]. During early tumor development, OPG works by interacting with TNF-Related Apoptosis-Inducing Ligand (TRAIL; TNFSF10), another cytokine that is known to promptly induce apoptosis in cell lines. The interaction between OPG and TRAIL blocks the process of apoptosis in tumor cells enhancing their survival [121,122] Gene modifications in OPG as well as its target RANKL has previously been conducted in animal models using the CRISPR/Cas9 system for research pertaining to bone defects [123] and perinatal brain injury [124]. More recently, in vitro studies involving OPG gene KO with the help of CRISPR/Cas9 have also been accomplished in MCF-7 breast cancer cell lines. OPG KO has been shown to inhibit the protein expression of Fatty Acid Synthase (FASN), an essential enzyme that is involved in the fatty acid biosynthetic pathway and is vital for breast cancer existence [125].

#### 3.2.7. UBR5

The Ubiquitin-Proteasome System (UPS) is a crucial regulator of cell signaling and proteostasis essential for cellular processes such as protein catabolism, apoptosis and cell cycle progression. Ubiquitination is a common post-translational modification (PTM) that involves activation, conjugation and ligation through the action of E1 ubiquitin-activating enzymes (E1), E2 ubiquitin-conjugating enzymes (E2) and E3 ubiquitin-ligase (E3), respectively in a sequential manner [126]. E3 Ubiquitin ligase holds a key position within the UPS as it recruits an E2 ubiquitin-conjugating enzyme and catalyzes the ubiquitination of a protein substrate. Such conjugated proteins may take part in oncogenesis [127]. 

BR5, a member of the E3 ligase family, has been overexpressed in the TNBC subtype unlike luminal A and B subtypes. Consequently, there may be a correlation between advanced clinical cancer stages with the expression of UBR5. Nonetheless, a successful knockout of the UBR5 in 4T1 and B16 cells was attained and showed characteristic morphological changes adapted by the epithelial cells into the mesenchymal shape that aids in the EMT. On the contrary, E-cadherin expression was entirely abolished in 4T1 cells devoid of UBR5. Since this protein plays a role in acquiring the invasive properties during EMT and assists in the advancement of late-stage breast cancer, its loss impairs the Mesenchymal-Epithelial Transition (MET) and hence the ability to settle into secondary organs. Additionally, decreased angiogenesis was observed in histological findings of the 4T1/ubr5-/- tumor 8 days post its inoculation. In short, the UBR5 gene plays a role in the enhancement of initial phases of tumor migration and invasion while limiting the later steps of metastatic colonization [128]. 

**Figure 4:**
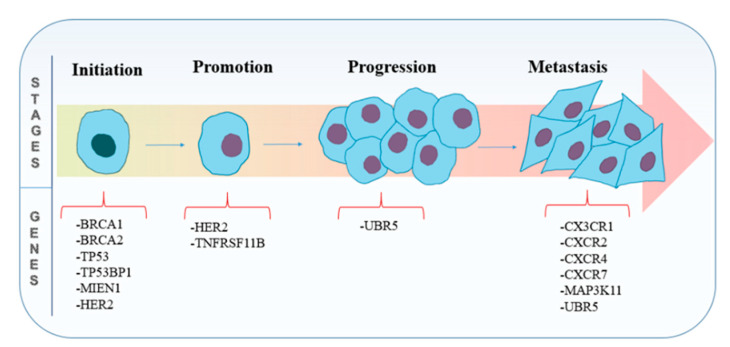
The different stages involved in carcinogenesis of breast cancer with respect to some of the contributing genes.

The different stages involved in the carcinogenesis of breast cancer with respect to some of the contributing genes are illustrated in Figure 4, and an overview of the potential therapeutic targets for breast cancer studied in literature employing the CRISPR/Cas9 technology is represented in Table 2.

## 4. Delivery Methods for CRISPR/Cas9

As promising and revolutionary the CRISPR/Cas9 system is, several challenges have been met especially with regards to its in vivo delivery due to its off-target effects [139]. Moreover, limited vector capacity and the size of CRISPR/Cas9 components further complicate this problem [140]. Delivery formats involve the use of plasmids, mRNA, ribonucleoprotein complexes (RNPs) or viral vectors [141]. Viral methods make up a large portion of the delivery methods for the CRISPR/Cas9 system to such an extent that delivery systems are usually classified as either ‘viral’ or ‘non-viral’ [142]. The most common delivery systems currently in use are discussed below.

### 4.1. Viral Delivery

Viral vectors, although posing problems like insertional mutagenesis and immunogenicity, remain the favored method for their high efficiency [143]. The genome-editing nuclease or genetic material is packaged into the viral vector and is then carried into the desired target cells. Various vectors have been employed including adenoviral vectors, lentiviral vectors and adeno-associated viral vectors [144].

#### 4.1.1. Adenoviruses (AdVs)

AdVs are a group of non-enveloped, double-stranded DNA viruses [145]. Since their discovery as gene delivery vectors, they have garnered a lot of interest and were among the first in vivo systems to be introduced [146]. These vectors, which are commonly extracted from human AdV5, do not integrate into the genome of the host but rather remain extrachromosomal, limiting the risk of insertional mutagenesis [147]. AdV delivery systems have shown to have an outstanding carrying capacity [148] and excellent transduction rates. However, they have also displayed the propensity to elicit a strong immune response in animal models [149]. Newer adenoviral generations void of all viral coding genes known as High Capacity Adenoviral Vectors (HC-AdVs) provide a more stable expression and larger carrying capacity. However, complicated production techniques remain a great obstacle [150].

#### 4.1.2. Lentiviruses (LVs)

LVs are single-stranded RNA viruses belonging to the retrovirus group. LV-based delivery allows concurrent delivery of Cas9 and gRNA due to its high packaging capacity [144]. The biggest advantage of lentiviral delivery is its high infection efficiency, even in quiescent cells [151]. Although its performance in assimilating into the host genome is exceptional, the non-specificity associated with this process presents the risk of random incorporation thus increasing the likelihood of insertional mutagenesis by integration into essential host genes. This may eventually result in carcinogenesis in severe cases [142]. A group of Integration-Deficient Lentiviral Vectors (IDLVs) has also been developed by specific integrase mutations and was shown to be linked with significantly less risk of insertional mutagenesis as well as generating replication-competent recombinants (RCRs) [152].

#### 4.1.3. Adeno-Associated Viruses (AAVs)

The AAV delivery system is one of the most popular viral delivery systems. Its long-lasting transgene expression, mild immune response, impressive infection potency and overall safety profile makes it a favorable approach [153]. The popularity of this system is augmented by the fact that AAV has already been approved in humans in other gene-therapy systems, and compared to other viruses, they have considerably lower immunogenic potential [154]. Furthermore, AAV systems offer controlled incorporation of CRISPR genes into mammalian cells, specifically the AAVS1 (adeno-associated virus integration site 1) locus and versatility in that they can transfect cells regardless of their mitotic phase [155]. The random-integration-related toxicity usually observed in other viral methods is circumvented by this precise delivery into the cell, deeming it the safest of the viral delivery methods and the most suitable for in vivo use [156]. Despite all its perks, the AAV system does have a carrying capacity limitation of about 4.7 kb and with the Cas9 protein alone being 4.3 kb, this hampers its therapeutic potential [142,157]. A summary of the viral delivery methods for CRISPR/Cas9 is represented in Table 3.

### 4.2. Non-Viral Delivery

Non-viral delivery systems may be further classified into (a) chemical methods such as Lipid mediated (Liposomal) or Calcium Phosphate transfection, and (b) physical methods including electroporation or microinjection. The main benefits of non-viral vectors are their ability to carry larger delivery components, their benign nature and convenient production [144]. Non-viral methods are generally capable of delivering genetic material into cells; however, this largely results in transient expressions and diminished efficiency which vary based on cell type [158].

#### 4.2.1. Electroporation

The electroporation method involves creating pores in the cell by the use of an electric current. These pores facilitate the entry of foreign DNA into the cytoplasm. Unlike viral-mediated approaches, this system does not have the same size restrictions and allows diverse DNA delivery [159]. Conversely, due to the high currents used, this method has also been shown to cause significant cell death [160].

#### 4.2.2. Microinjection

Microinjection is a simple but difficult to apply technique of incorporating exogenous DNA or protein into a cell and over the years has become a standard laboratory procedure. During this process, the direct transfer of genetic components is carried out using a micropipette. The narrow tip of these micropipettes, combined with the high precision of the micromanipulators that are used, allow for transfection that is both reliable and precise [161]. This method possesses a degree of considerable specificity and reproducibility and is suitable for delivering all CRISPR/Cas9 formats. On the contrary, it may have poor throughput, causing cellular damage thus demanding proficiency in manual skills [151].

#### 4.2.3. Calcium Phosphate Delivery

The basis of this method is DNA condensation that is achieved by blending the DNA with calcium phosphate, which is not only involved in forming a complex but also aids in cell surface adhesion. The cell then engulfs this complex by means of endocytosis, facilitating the release of the genetic material inside the cell [162]. This approach has been around for decades because of the relative affordability and abundance of the reagents required. On the other hand, its high toxicity in some cells makes it inappropriate in specific cases. Furthermore, condition optimization and monitoring are key as slight changes may jeopardize transfection levels [163].

#### 4.2.4. Lipid-Mediated Delivery

This approach, also known as liposomal delivery, involves the interaction of positively charged cationic lipids with the phosphate groups in foreign DNA to form liposomes [164]. The resulting liposomes then fuse with the cell membrane to gain entry into the target cell and subsequently transfer the genetic material. This method has both exceptional transfection rates and versatility in that various cell lines may be utilized; however, this is largely dependent on culture conditions [165]. A summary of the non-viral delivery methods for CRISPR/Cas9 is represented in Table 4.

### 4.3. Targeted Delivery

Despite significant progress in the development of the CRISPR/Cas9 platform, in vivo delivery, whether systemic or targeted, remains one of the largest barriers thus far. The goal for in vivo delivery is to increase the CRISPR concentration towards the target tissue and to decrease any unwanted effects on the rest of the body. There are two key elements in developing the CRISPR/Cas system for clinical application. To start with, the system should be able to target the desired cells selectively. Moreover, when the components of CRISPR are taken in, it is important that they evade the harsh physiologic environment to obtain entry to the core and achieve genetic modification [166]. Targeted delivery methods that have been implemented usually involve the recognition of ligands by cell-specific receptors that induce endocytosis. One example of this was observed in a human hepatocarcinoma cell line, whereby the researchers found evidence of liver cell-specific internalization and thus EMX1 gene editing was achieved [167]. Nonetheless, this technique not only involved complex Cas 9 protein alterations but also relied on the use of endosomolytic peptides to help them escape as cells did not have a means of protecting themselves from enzymatic degradation [167,168,169]. Additionally, the creation of a selective organ targeting (SORT) system that has been shown to be compatible with both the CRISPR/Cas sgRNA as well as RNP complexes is another particularly impressive approach. This technique involves changing the core charge of lipid nanoparticle transporters by changing the composition of a supplementary ‘SORT’ molecule [170]. Researchers have used this technique to demonstrate lung-, liver- and spleen-specific targeting in mice, as well as effective gene editing of PTEN, which is a known therapeutic target for cancer [170]. Alternative delivery methods comprise of exploiting the homotypic binding phenomenon that is innately seen in tumors to target homotypic tumors by using nanoparticles that were coated with Cas9 RNPs that had MCF-7 cancer cell membranes [171].

## 5. Clinical Trials

The CRISPR/Cas9 system holds great potential in treating a wide variety of human diseases ranging from genetic disorders to cancer. In a study published in January 2021, the BCLA11 gene, responsible for repressing fetal hemoglobin production, was deleted in CD34+ hematopoietic stem and progenitor cells utilizing CRISPR/Cas9 via electroporation. Two patients—one with transfusion-dependent β-thalassemia (TDT) and sickle cell disease (SCD)—received autologous CRISPR/Cas9 edited CD4+ cells; a year later, both patients showed great results with an increase in fetal hemoglobin pancellularly distributed and transfusion independence [172]. Such a study shows support for the effectiveness of CRISPR/Cas9 for its potential application in disease treatment. With regards to breast cancer, as of April 2021, no clinical trials have been conducted. However, trials are currently underway for other types of cancer such as B-cell acute lymphoblastic leukemia. As we have discussed above, the promising results of employing CRISPR/Cas9 in preclinical studies of genes involved in breast cancer could potentiate the start of a clinical trial. Moreover, CRISPR/Cas9 can be integrated as part of chemotherapeutics to target the genes discussed in context with not only breast cancer but also other malignancies, if ever the contributing genes were to be mutated. Although the use of the CRISPR/Cas9 system is not yet widespread, the increasing number of preclinical studies could provide better insight into the system and genetic etiology of varying disease conditions, which could contribute to the application of CRISPR/Cas9 in the clinical setting. Table 5 shows examples of ongoing clinical trials employing CRISPR/Cas9 for various types of cancer as listed on clinicaltrials.gov.

## 6. Implications for the Future

### 6.1. Precision Genome Editing

As evidenced by multiple research works, CRISPR/Cas9 has immense potential for editing genomes to precision. In a paper published in 2013, TALENs and the CRISPR were studied for their relative target efficiencies by keeping the genomic targets in the hPSC lines and the delivery methods for the systems as a control. The findings suggested that CRISPR was able to outperform TALENs by around 45–51%; yielding mutant clones at estimated efficiencies of 51–79% [173]. Additionally, because polyploid cancer cells harnessing unstable genomes pose a challenge for precision editing, another study focused on its knock-in strategies using CRISPR/Cas9. Site-specific lysine 295 (K295) was substituted for glutamine (K295Q) in FOXA1, a critical transcription factor involved in the signaling of the estrogen and androgen receptors in MCF-7 Luminal A breast cancer cells. TOPO cloning allele frequency data and RNA- seq of these cell lines resulted in finding 28–100% mature KI mutation-carrying mRNA. This concludes that CRISPR/Cas9 can facilitate systemic analysis of the molecular mechanisms involved in various breast cancer types, with the utmost precision [174].

### 6.2. Mapping Genetic Interactions

Precision in breast cancer therapeutics, diagnosis and prognosis calls for precise therapeutic targets in this heterogenic natured cancer. To elaborate on the cell-specific mechanisms of drug actions, a computational target deconvolution pipeline was developed in a study, where key target dependencies would be identified based on patterns of pooled drug response in each of the cell lines used. Drug–cell line responses were combined quantitatively by the pipelines with drug–target interactions to identify effective and selective therapeutic targets. The study applied 310 small molecules to the target deconvolution pipeline on 20 heterogeneous TNBC cell lines. The Target Addiction Score (TAS) was used to quantify the functionality of each protein by measuring the dependency of the cell line on the therapeutic target. The therapeutic targets were selected through CRISPR-mediated knockouts, chemical proteomics and other experimental methods. Additionally, gene essentiality scores can improve predictions of cancer dependency maps [175] and can be obtained from CRISPR/Cas9 knockout screens and TAS profiles, which denotes that some proteins with low gene essentiality exhibit high target addictions, functioning as protein groups and hence being resistant to single-gene knock-out. Furthermore, histone deacetylases and cyclin-dependent kinases were recovered using this approach. This provides a broader insight into the druggable targets of TNBC and hints at further studies that can identify multi-target synthetic lethal interactions [174].

### 6.3. Synthetic Lethality Screens

CRISPR/Cas9 has been credited for identifying a kinetochore-microtubule-dependent mechanism in a study that looked into the reason behind a selective Aurora-A inhibitor alisertib (MLN8237) drug candidate failing its phase III clinical trials. Overexpression of Aurora-A kinases is linked with poor prognosis in breast cancer patients. CRISPR/Cas9 mediated systematic synthetic lethality screening of 507 kinases using alisertib in breast cancer cells—with 10 sgRNAs guiding per kinase—was performed and targetable kinases with synthetic lethality interactions with alisertib were identified by distinguishing genes that were able to enhance alisertib sensitivity upon their inhibition or deletion in breast cancer cells. Synthetic lethal interactions were observed between multiple depletions of multiple kinases. Several assays were performed to assess the synergistic effects that arose due to Haspin inhibition with alisertib. Further analysis indicated that Haspin inhibition enhanced the lethal effects of alisertib but was able to inhibit breast cancer cell growth only slightly. However, combining a Haspin inhibitor (CHR-6494) with alisertib reduced cell viability favorably and inhibited its in vivo and in vitro tumor growth by causing extreme microtubule depolymerization, and along with other mechanisms, the mitotic process was disrupted, brought forth by the obliteration of the recruitment of Aurora-B and mitotic centromere associated kinesin to the centromeres. This ultimately demonstrates the use of CHR-6494 as a combinational drug with alisertib and its promising therapeutic benefits [176]. Future implications of breast cancer genes studied using CRISPR/Cas9 are summarized in Table 6.

## 7. Limitations

### 7.1. Off-Target Effects

Studies have reported that Cas9 has the ability to unintentionally bind to unwanted genomic sites for cleavage, leading to an off-target effect (OTE) which has the potential to result in a mutation that could be consequential. This is a crucial hindrance in the implementation of CRISPR/Cas9 as a clinical platform not only for breast cancer treatment but gene therapy in general. However, attempts at rectifying this issue include the use of Cas9 variants engineered to reduce the OTE and optimization of the sgRNA. An example of a Cas9 variant designed to minimize the OTE is the Cas9 nickase (Cas9n) which induces a single-stranded break, used together with the sgRNA to target both strands of the intended DNA sequence to deliver the DSB [177]. A high-fidelity variant of Cas9, termed SpCas9-HF1, has also been engineered. An ‘excess-energy’ model that demonstrates an excessive affinity between Cas9 and the target DNA has been proposed to be one of the reasons for OTE. SpCas9-HF1 makes use of this excess energy. Mutations were introduced to four residues directly involved in the hydrogen binding between Cas9 and the target DNA phosphate backbone, results indicated that no OTE was traced with SpCas9-HF1 compared to SpCas9 wildtype [178]. The sgRNA determines specificity to the target DNA sequence, hence optimization of the sgRNA could be another means of reducing the occurrence of OTE. Recently, a guide design tool called sgDesigner was developed; it utilizes a novel plasmid library in silico that contains the sgRNA and target site within the same setup, enabling the collection of Cas9 editing efficiency data intrinsically. Moreover, a performance evaluation between sgDesigner and other frequently used tools (DeepCRISPR, Sequence Scan for CRISPR and Doench Rule Set 2) to predict the efficiency of sgRNA indicated that the sgDesigner outperformed the other designer tools, suggesting the sgDesigner may be a more reliable tool [179].

### 7.2. Protospacer Adjacent Motif Requirement

The requirement of a PAM sequence directly after the target binding site of crRNA is another limitation of the CRISPR system. SpCas9, the most commonly used CRISPR/Cas9 system, recognizes a short PAM sequence 5′-NGG-3′, where N is any nucleotide. Despite the need for a relatively short PAM sequence, it still constrains the possible target sites leaving numerous sequences inaccessible to genome editing. A SpCas9 variant, termed SpRY, was recently engineered to be virtually PAMless. SpRY exhibits editing activity with minimal recognition of NRN PAM over NYN PAM. This new SpCas9 variant could have promising applications in gene editing independent of a PAM sequence requirement [180]. 

### 7.3. Delivery

The safety and therapeutic efficacy of CRISPR are largely dependent on the delivery techniques available. Conventional gene therapies that employ viral vectors have faced backlash for the risk of insertional mutagenesis and immune reactions. Nevertheless, these viral vectors remain a key delivery tool for CRISPR therapy and are still extensively used for their high-efficiency rates. One technique that has greatly reduced some of the OTEs without compromising efficacy is delivering the components of this system as RNP complexes [181]. CRISPR/Cas9 edits can be carried out either ex vivo by gene modification externally and then re-injection back into the patient, or in vivo by delivering the components of CRISPR into the patient directly. With either method, there come benefits and some drawbacks. Ex vivo methods offer a more impressive safety profile and more rigorous control in the quality of cells [182]. On the other hand, the therapeutic potential of ex vivo methods is drastically hindered due to culture conditions that have proven inappropriate for use in a majority of tissues with the exception of T cells and hematopoietic stem and progenitor cells. For that reason, in certain hematological diseases and cancers, ex vivo delivery may be applicable [183]. In vivo manipulation also faces a number of obstacles, physiologically including, escaping degradation by hydrolysis or opsonization, circumventing intricate vascular systems and actually reaching the target tissue. Furthermore, confinement of the delivery contents at the site of injection may lead to unequal distribution and as a result substandard responses to therapy [184]. Although continuous improvements are being carried out, as of right now, most trials involve patients with serious diseases only [185]. Continuous application of this system and conclusions from trials may prove to be important deciding factors in how we integrate this system into the healthcare field moving forward. Critics argue whether condoning CRISPR/Cas9 editing and other gene therapies may serve as a doorway for non-therapeutic and more trivial objectives, such as the case in China with the CRISPR-baby scandal [185].

### 7.4. Ethical Consideration

In 2018, He Jiankui made headlines around the world for revealing the birth of the first-ever gene-edited babies in China. The twin girls were claimed to have their CCR5 gene disabled using the CRISPR technology in hopes to resist potential HIV, smallpox and cholera infections throughout their lives. However, this quickly became a matter of ethical concern. Whether this could have repercussions in the long run for the human subjects or would mutations with unknown consequences pass on to subsequent generations, violating their rights, sparked considerable debate [186]. 

This is, for the most part, due to the lack of fully understanding the relationship between genetic information and its corresponding phenotypes. Moreover, not only do the vast intricacies of regulatory mechanism for multiple genes determine biological traits but epigenetics, regulatory elements, additional genes and environmental factors also play a role. Similarly, although preclinical studies involving CRISPR have the potential to transition from bench to bedside, careful consideration is needed to prevent unanticipated side effects ranging from safety in terms of the tool’s on/off-target efficiency to the ethical and moral concerns of transgenerational risks [187]. 

The coupled potential of CRISPR as the future of gene editing and its unknown risks calls for evidence-based decision-making. Such ethical conundrums can be addressed if strictly regulated genetic experiments and clinical trials are permitted without major restrictions by governing bodies dictating laws on funding and research [188]. Extensive data on the benefits and risks would thereupon aid in minimizing and maximizing the potential of the technology’s risks and benefits respectively.

## 8. Conclusions

The CRISPR/Cas9 system has undoubtedly made its mark in the field of science through its efficiency and versatility in gene editing compared to its earlier predecessors. This Nobel Prize winning platform has displayed immense clinical potential through its use not only in the detection but also in the screening of therapeutic targets for genetic diseases which are notoriously difficult to treat due to the lack of definitive therapies. A prime example of this is the employment of CRISPR/Cas9 in studies involving breast cancer, where causal relationships have been established with specific genetic mutations. In most cases, manipulation of genes using CRISPR/Cas9 has led to significant suppression in various stages of tumorigenesis including initiation, progression, proliferation and metastasis. However, despite all the traction it has gained, limitations with regards to its ethics, off-target effects, mutagenesis and delivery necessitate further studies to rectify these issues. For the conventional use of this system in the near future, both precise knowledge of pathogenic variants, as well as optimization of the system itself, is essential to venture into the new era of personalized medicine.

## Figures and Tables

**Table 1:** Classification of the CRISPR/Cas Systems.

CRIPSR/Cas Systems						
Class	1			2		
Protein type	Multiplex			Single		
Type	I	III	IV	II	V	VI
Corresponding Cas protein	Cas 3	Cas 10	Cas 8	Cas 9	Cas 12a, Cas 12c, Cas 13a	Cas 13b, Cas 13c

**Table 2:** An overview of the potential therapeutic targets for breast cancer studied in literature employing the CRISPR/Cas9 technology.

Gene	Protein Encoded	Role in Breast Cancer	Associated Subtype of Breast Cancer	CRISPR/Cas9 Application	Results	Ref.
BRCA 1	BRCA1	Lacks DSB repair ability	TNBC	Nucleotide substitution through BE	Successful identification of LOF variants	[41,47,129]
BRCA 2	BRCA2	Lacks DSB repair ability	ER+ and HER2-	KO	Cell inviability	[51,53,129]
HER2	HER2	Promotes cell proliferation	HER2+	KO	Suppressed cell proliferation and tumorigenesis	[55]
TP53	p-53	Deregulates cell cycle	TNBC and/or HER2+	Cas9 expressed in cell lines	Cas9 induces TP53 mutation	[62,63,130]
TP53BP1	53BP1	Lacks DSB repair ability	TNBC	KO	Restoration of HR in BRCA1 and P53BP1 deficient cells	[64,131]
MKI67	Ki-67	Promotes initiation, progression, metastasis	N/A	KO	Wide-spread transcriptome changes	[71]
MIEN1	MIEN1 protein	Promotes initiation, metastasis	TNBC	KO	No effect on cell viability and tumor proliferation	[92]
CX3CR1	CX3CR1	Promotes skeletal and soft tissue organ metastasis	Luminal A&B, HER2+, TNBC	Transcription silencing	Reduction in skeletal and lung metastasis	[93]
CXCR2	CXCR2	Promotes cell proliferation, migration, angiogenesis	TNBC	KO	Suppressed cell proliferation, migration and metastasis	[104]
CXCR4 and CXCR7	CXCR4 and CXCR7	Promotes cell proliferation, invasion, metastasis	TNBC	Single-gene knockout and co-knockout	Suppressed cell proliferation, invasion and migration	[106]
MAP3K11	MLK 3	Promotes metastasis	TNBC	KO	Suppressed cell invasion and migration	[118]
TNFRSF11B	OPG	Blocks apoptosis	HR+	KO	Inhibited protein expression of Fatty Acid Synthase	[125]
UBR5	Ubr5	Aids EMT	TNBC	KO	Induced apoptosis and suppressed metastasis	[128]
CDK4	CDK4	Cell proliferation	TNBC and HER2+	KO	Suppressed viability, clono-genicity, migration	[132]
MFN2	MFN2	Suppress cancer progression via mTOR2/Akt signal inhibition	HR+	KO	Promotes cell viability, invasion, colony formation	[133]
APOBEC3G	APOBEC3	APOBEC3 induced mutagenesis	HER2+	KO	Suppressed cell proliferation	[134]
MARK4	MARK4	Inhibits Hippo signaling leading to cell proliferation	TNBC	KO	Suppressed cell proliferation and migration	[135]
MASTL	MASTL	Promotes proliferation	HR+ and TNBC	KO	Suppressed proliferation and tumor growth	[136]
MELK	MELK	Tumorigenesis regulator	TNBC	KO	CRISPR/Cas9-Mediated Mutagenesis	[137]
MFGE8	MFGE8	Mediator of breast cancer tumorigenesis	TNBC	KO	Restored sensitivity to COX-2 selective inhibitor	[138]
KLK5	KLK5	Serum biomarker	TNBC	KO		
KLK7	KLK7					

**Table 3:** Summary of the viral delivery methods for CRISPR/Cas9 (+: Low; ++: Moderate; +++: High)

Vector	Mechanism	Immunogenicity	Packaging Capacity (kb)	Reference
Lentivirus	Incorporates into host genome	++	9.7	[144]
Adenovirus	Do not integrate; remain extrachromosomal	+++	35	[148]
Adeno-mediated adenovirus	May incorporate; primarily remain extrachromosomal	+	4.7	[142,157]

**Table 4:** Summary of the non-viral delivery methods for CRISPR/Cas9.

Method	Mechanism	Advantage	Disadvantage	Reference
Electroporation	Creates pores in plasma membrane; increases permeability	No size restrictions	High cellular lysis	[158]
Microinjection	Ejection of genetic material using a micro-pipette	Good reproducibility	Lower throughput	[151]
Calcium Phosphonate	Blending genetic material with CaPO4 improves cellular adhesion and entry	Affordable	High cytotoxicity	[163]
Lipid-mediated	Liposomal fusion with cell membrane to facilitate access into cell	High transfection rates	Culture condition affect efficiency	[165]

**Table 5:** Table that summarizes the clinical trials employing CRISPR/Cas9 for various types of cancer.

Cancer	Phase	Intervention	Country	ID
Gastrointestinal, Epithelial Cancer, Gastrointestinal Neoplasms, Cancer of Gastrointestinal Tract, Cancer, Gastrointestinal, Gastrointestinal Cancer, Colo-rectal Cancer, Pancreatic Cancer, Gall Bladder Cancer, Colon Cancer, Esophageal Cancer, Stomach Cancer	I/II	Drug: Cyclophosphamide Drug: Fludarabine Biological: Tumor-Infiltrating Lymphocytes (TIL) Drug: Aldesleukin	USA	NCT04426669
Solid Tumor, Adult	I	Biological: anti-mesothelin CAR-T cells	China	NCT03545815
B-cell Malignancy, Non-Hodgkin Lymphoma, B-cell Lymphoma, Adult B Cell ALL	I	Biological: CTX110	USA	NCT04035434
T Cell Lymphoma	I	Biological: CTX130	USA	NCT04502446
Multiple Myeloma	I	Biological: CTX120	USA	NCT04244656
B Cell Leukemia, B Cell Lymphoma	I/II	Biological: UCART019	China	NCT03166878
Renal Cell Carcinoma	I	Biological: CTX130	USA	NCT04438083
B Acute Lymphoblastic Leukemia	I	Drug: PBLTT52CAR19	UK	NCT04557436
B Cell Leukemia, B Cell Lymphoma	I/II	Biological: Universal Dual Specificity CD19 and CD20 or CD22 CAR-T Cells	China	NCT03398967

**Table 6:** Future implications of breast cancer genes studied using CRISPR/Cas9.

Gene Studied	Prospective Application
BRCA1	Reclassification of BRCA1 and potentially other breast cancer VUSs through CRISPR-based high-throughput screens.
BRCA2	Further studies are needed to understand how knock-out of BRCA2, a tumor suppressor, leads to cell inviability, yet this is overcome in BRCA2 mutated breast cancers.
HER2	CRISPR/Cas9 can be used to target HER2 to achieve a therapeutic outcome in clinical settings.
TP53	The study highlighted a limitation when conducting future experiments on TP53 with Cas9.
TP53BP1	PALB2 could be tested for its clinical implications in clinically significant BRCA1/53BP1 deficient cells.
MKI67	Further studies on identifying the interacting proteins and effectors involved in MKI67 knock-out can help identify therapeutic targets.
CXCR4 and CXCR7	These genes can function as possible therapeutic targets for TNBC treatment.
MAP3K11	This gene can function as a possible therapeutic target for TNBC treatment.
CX3CR1	Can be targeted to limit breast cancer metastasis to the bone.
CXCR2	This gene can function as a possible therapeutic target for TNBC treatment.
UBR5	This gene can function as a possible therapeutic target for TNBC treatment.
MIEN1	Further studies are required on this gene to substantiate the function of MIEN1 in metastasis.

## Data Availability

Not applicable.

## References

[1] Ishino Y., Shinagawa H., Makino K., Amemura M., Nakata A. (1987). Nucleotide sequence of the iap gene, responsible for alkaline phosphatase isozyme conversion in Escherichia coli, and identification of the gene product. J. Bacteriol..

[2] Bray F., Ferlay J., Soerjomataram I., Siegel R.L., Torre L.A., Jemal A. (2018). Global cancer statistics 2018: GLOBOCAN estimates of incidence and mortality worldwide for 36 cancers in 185 countries. CA Cancer J. Clin..

[3] Hortobagyi G.N., de la Garza Salazar J., Pritchard K., Amadori D., Haidinger R., Hudis C.A., Khaled H., Liu M.-C., Martin M., Namer M. (2005). The Global Breast Cancer Burden: Variations in Epidemiology and Survival. Clin. Breast Cancer.

[4] Makarova K.S., Haft D.H., Barrangou R., Brouns S.J.J., Charpentier E., Horvath P., Moineau S., Mojica F.J.M., Wolf Y.I., Yakunin A.F. (2011). Evolution and classification of the CRISPR—Cas systems. Nat. Rev. Microbiol..

[5] Makki J. (2015). Diversity of Breast Carcinoma: Histological Subtypes and Clinical Relevance. Clin. Med. Insights Pathol..

[6] Akram M., Iqbal M., Daniyal M., Khan A.U. (2017). Awareness and current knowledge of breast cancer. Biol. Res..

[7] Ma Y., Zhang L., Huang X. (2014). Genome modification by CRISPR/Cas9. FEBS J..

[8] Hille F., Richter H., Wong S.P., Bratovič M., Ressel S., Charpentier E. (2018). The Biology of CRISPR-Cas: Backward and Forward. Cell.

[9] Mojica F.J.M., Diez-Villasenor C., Garcia-Martinez J., Soria E. (2005). Intervening Sequences of Regularly Spaced Prokaryotic Repeats Derive from Foreign Genetic Elements. J. Mol. Evol..

[10] Cong L., Ran F.A., Cox D., Lin S., Barretto R., Habib N., Hsu P.D., Wu X., Jiang W., Marraffini L.A. (2013). Multiplex Genome Engineering Using CRISPR/Cas Systems. Science.

[11] Arnedos M., Vicier C., Loi S., Lefebvre C., Michiels S., Bonnefoi H., Andre F. (2015). Precision medicine for metastatic breast cancer—limitations and solutions. Nat. Rev. Clin. Oncol..

[12] Khan S.H. (2019). Genome-Editing Technologies: Concept, Pros, and Cons of Various Genome-Editing Techniques and Bioethical Concerns for Clinical Application. Mol. Ther. Nucleic Acids.

[13] Salsman J., Dellaire G. (2017). Precision genome editing in the CRISPR era. Biochem. Cell Biol..

[14] Zhang J.-H., Adikaram P., Pandey M., Genis A., Simonds W.F. (2016). Optimization of genome editing through CRISPR-Cas9 engineering. Bioengineered.

[15] Riehle M.M., Bennett A.F., Long A.D. (2001). Genetic architecture of thermal adaptation in Escherichia coli. Proc. Natl. Acad. Sci. USA.

[16] DeBoy R.T., Mongodin E.F., Emerson J.B., Nelson K.E. (2006). Chromosome Evolution in the Thermotogales: Large-Scale Inversions and Strain Diversification of CRISPR Sequences. J. Bacteriol..

[17] Jansen R., van Embden J.D.A., Gaastra W., Schouls L.M. (2002). Identification of genes that are associated with DNA repeats in prokaryotes. Mol. Microbiol..

[18] Mojica F.J.M., Diez-Villasenor C., Soria E., Juez G. (2000). Biological significance of a family of regularly spaced repeats in the genomes of Archaea, Bacteria and mitochondria. Mol. Microbiol..

[19] Pourcel C., Salvignol G., Vergnaud G. (2005). CRISPR elements in Yersinia pestis acquire new repeats by preferential uptake of bacteriophage DNA, and provide additional tools for evolutionary studies. Microbiology.

[20] Makarova K.S., Wolf Y.I., Iranzo J., Shmakov S.A., Alkhnbashi O.S., Brouns S.J.J., Charpentier E., Cheng D., Haft D.H., Horvath P. (2020). Evolutionary classification of CRISPR–Cas systems: A burst of class 2 and derived variants. Nat. Rev. Microbiol..

[21] Makarova K.S., Wolf Y.I., Alkhnbashi O.S., Costa F., Shah S.A., Saunders S.J., Barrangou R., Brouns S.J.J., Charpentier E., Haft D.H. (2015). An updated evolutionary classification of CRISPR—Cas systems. Nat. Rev. Microbiol..

[22] Jinek M., Chylinski K., Fonfara I., Hauer M., Doudna J.A., Charpentier E. (2012). A Programmable Dual-RNA-Guided DNA Endonuclease in Adaptive Bacterial Immunity. Science.

[23] Mali P., Yang L., Esvelt K.M., Aach J., Guell M., DiCarlo J.E., Norville J.E., Church G.M. (2013). RNA-Guided Human Genome Engineering via Cas9. Science.

[24] Barrangou R. (2013). CRISPR-Cas systems and RNA-guided interference: CRISPR-Cas systems and RNA-guided interference. WIREs RNA.

[25] Bhaya D., Davison M., Barrangou R. (2011). CRISPR-Cas Systems in Bacteria and Archaea: Versatile Small RNAs for Adaptive Defense and Regulation. Annu. Rev. Genet..

[26] Yosef I., Goren M.G., Qimron U. (2012). Proteins and DNA elements essential for the CRISPR adaptation process in *Escherichia coli*. Nucleic Acids Res..

[27] Nuñez J.K., Kranzusch P.J., Noeske J., Wright A.V., Davies C.W., Doudna J.A. (2014). Cas1–Cas2 complex formation mediates spacer acquisition during CRISPR–Cas adaptive immunity. Nat. Struct. Mol. Biol..

[28] Heler R., Samai P., Modell J.W., Weiner C., Goldberg G.W., Bikard D., Marraffini L.A. (2015). Cas9 specifies functional viral targets during CRISPR–Cas adaptation. Nature.

[29] Deltcheva E., Chylinski K., Sharma C.M., Gonzales K., Chao Y., Pirzada Z.A., Eckert M.R., Vogel J., Charpentier E. (2011). CRISPR RNA maturation by trans-encoded small RNA and host factor RNase III. Nature.

[30] Thurtle-Schmidt D.M., Lo T.-W. (2018). Molecular biology at the cutting edge: A review on CRISPR/CAS9 gene editing for undergraduates: A Review on CRISPR/CAS9 Gene Editing for Undergraduates. Biochem. Mol. Biol. Educ..

[31] Gasiunas G., Barrangou R., Horvath P., Siksnys V. (2012). Cas9-crRNA ribonucleoprotein complex mediates specific DNA cleavage for adaptive immunity in bacteria. Proc. Natl. Acad. Sci. USA.

[32] Anders C., Niewoehner O., Duerst A., Jinek M. (2014). Structural basis of PAM-dependent target DNA recognition by the Cas9 endonuclease. Nature.

[33] Jinek M., Jiang F., Taylor D.W., Sternberg S.H., Kaya E., Ma E., Anders C., Hauer M., Zhou K., Lin S. (2014). Structures of Cas9 Endonucleases Reveal RNA-Mediated Conformational Activation. Science.

[34] Nishimasu H., Ran F.A., Hsu P.D., Konermann S., Shehata S.I., Dohmae N., Ishitani R., Zhang F., Nureki O. (2014). Crystal Structure of Cas9 in Complex with Guide RNA and Target DNA. Cell.

[35] Rodriguez-Rodriguez D., Ramirez-Solis R., Garza-Elizondo M., Garza-Rodriguez M., Barrera-Saldana H. (2019). Genome editing: A perspective on the application of CRISPR/Cas9 to study human diseases (Review). Int. J. Mol. Med..

[36] Biagioni A., Chillà A., Andreucci E., Laurenzana A., Margheri F., Peppicelli S., Del Rosso M., Fibbi G. (2017). Type II CRISPR/Cas9 approach in the oncological therapy. J. Exp. Clin. Cancer Res..

[37] Jiang F., Doudna J.A. (2017). CRISPR–Cas9 Structures and Mechanisms. Annu. Rev. Biophys..

[38] Wyman C., Kanaar R. (2006). DNA Double-Strand Break Repair: All’s Well that Ends Well. Annu. Rev. Genet..

[39] Doench J.G., Fusi N., Sullender M., Hegde M., Vaimberg E.W., Donovan K.F., Smith I., Tothova Z., Wilen C., Orchard R. (2016). Optimized sgRNA design to maximize activity and minimize off-target effects of CRISPR-Cas9. Nat. Biotechnol..

[40] Martinez-Lage M., Puig-Serra P., Menendez P., Torres-Ruiz R., Rodriguez-Perales S. (2018). CRISPR/Cas9 for Cancer Therapy: Hopes and Challenges. Biomedicines.

[41] Petrucelli N., Daly M.B., Feldman G.L. (2010). Hereditary breast and ovarian cancer due to mutations in BRCA1 and BRCA2. Genet. Med..

[42] Huen M.S.Y., Sy S.M.H., Chen J. (2010). BRCA1 and its toolbox for the maintenance of genome integrity. Nat. Rev. Mol. Cell Biol..

[43] Brzovic P.S., Rajagopal P., Hoyt D.W., King M.-C., Klevit R.E. (2001). Structure of a BRCA1-BARD1 heterodimeric RING-RING complex. Nat. Struct. Biol..

[44] Brzovic P.S., Keeffe J.R., Nishikawa H., Miyamoto K., Fox D., Fukuda M., Ohta T., Klevit R. (2003). Binding and recognition in the assembly of an active BRCA1/BARD1 ubiquitin-ligase complex. Proc. Natl. Acad. Sci. USA.

[45] Yamane K., Katayama E., Tsuruo T. (2000). The BRCT Regions of Tumor Suppressor BRCA1 and of XRCC1 Show DNA End Binding Activity with a Multimerizing Feature. Biochem. Biophys. Res. Commun..

[46] Liu J., Pan Y., Ma B., Nussinov R. (2006). “Similarity Trap” in Protein-Protein Interactions Could Be Carcinogenic: Simulations of p53 Core Domain Complexed with 53BP1 and BRCA1 BRCT Domains. Structure.

[47] Kweon J., Jang A.-H., Shin H.R., See J.-E., Lee W., Lee J.W., Chang S., Kim K., Kim Y. (2020). A CRISPR-based base-editing screen for the functional assessment of BRCA1 variants. Oncogene.

[48] Corso G., Feroce I., Intra M., Toesca A., Magnoni F., Sargenti M., Naninato P., Caldarella P., Pagani G., Vento A. (2018). BRCA1/2 germline missense mutations: A systematic review. Eur. J. Cancer Prev..

[49] Roy R., Chun J., Powell S.N. (2012). BRCA1 and BRCA2: Different roles in a common pathway of genome protection. Nat. Rev. Cancer.

[50] Suwaki N., Klare K., Tarsounas M. (2011). RAD51 paralogs: Roles in DNA damage signalling, recombinational repair and tumorigenesis. Semin. Cell Dev. Biol..

[51] Wong M.W., Nordfors C., Mossman D., Pecenpetelovska G., Avery-Kiejda K.A., Talseth-Palmer B., Bowden N.A., Scott R.J. (2011). BRIP1, PALB2, and RAD51C mutation analysis reveals their relative importance as genetic susceptibility factors for breast cancer. Breast Cancer Res. Treat..

[52] Prakash R., Zhang Y., Feng W., Jasin M. (2015). Homologous Recombination and Human Health: The Roles of BRCA1, BRCA2, and Associated Proteins. Cold Spring Harb. Perspect. Biol..

[53] Feng W., Jasin M. (2017). BRCA2 suppresses replication stress-induced mitotic and G1 abnormalities through homologous recombination. Nat. Commun..

[54] Slamon D., Clark G., Wong S., Levin W., Ullrich A., McGuire W. (1987). Human Breast Cancer: Correlation of Relapse and Survival with Amplification of the HER-2/neu Oncogene. Science.

[55] Wang H., Sun W. (2017). CRISPR-mediated targeting of HER2 inhibits cell proliferation through a dominant negative mutation. Cancer Lett..

[56] Gutierrez C., Schiff R. (2011). HER2: Biology, detection, and clinical implications. Arch. Pathol. Lab. Med..

[57] Yarden Y. (2001). Biology of HER2 and Its Importance in Breast Cancer. Oncology.

[58] Moasser M.M. (2007). The oncogene HER2: Its signaling and transforming functions and its role in human cancer pathogenesis. Oncogene.

[59] Home—My Cancer Genome. https://www.mycancergenome.org/.

[60] Welcome—IARC TP53 Database. https://p53.iarc.fr/.

[61] Zhu J., Sammons M.A., Donahue G., Dou Z., Vedadi M., Getlik M., Barsyte-Lovejoy D., Al-awar R., Katona B.W., Shilatifard A. (2015). Gain-of-function p53 mutants co-opt chromatin pathways to drive cancer growth. Nature.

[62] Levine A.J., Berger S.L. (2017). The interplay between epigenetic changes and the p53 protein in stem cells. Genes Dev..

[63] (2020). Metastasis Has Multiple Origins and Occurs Early in Tumorigenesis. Cancer Discov..

[64] Belotserkovskaya R., Raga Gil E., Lawrence N., Butler R., Clifford G., Wilson M.D., Jackson S.P. (2020). PALB2 chromatin recruitment restores homologous recombination in BRCA1-deficient cells depleted of 53BP1. Nat. Commun..

[65] Xia B., Sheng Q., Nakanishi K., Ohashi A., Wu J., Christ N., Liu X., Jasin M., Couch F.J., Livingston D.M. (2006). Control of BRCA2 Cellular and Clinical Functions by a Nuclear Partner, PALB2. Mol. Cell.

[66] Ripperger T., Gadzicki D., Meindl A., Schlegelberger B. (2009). Breast cancer susceptibility: Current knowledge and implications for genetic counselling. Eur. J. Hum. Genet..

[67] Wiltshire T., Ducy M., Foo T.K., Hu C., Lee K.Y., Belur Nagaraj A., Rodrigue A., Gomes T.T., Simard J., Monteiro A.N.A. (2020). Functional characterization of 84 PALB2 variants of uncertain significance. Genet. Med..

[68] Cidado J., Wong H.Y., Rosen D.M., Cimino-Mathews A., Garay J.P., Fessler A.G., Rasheed Z.A., Hicks J., Cochran R.L., Croessmann S. (2016). Ki-67 is required for maintenance of cancer stem cells but not cell proliferation. Oncotarget.

[69] Castéra L., Harter V., Muller E., Krieger S., Goardon N., Ricou A., Rousselin A., Paimparay G., Legros A., Bruet O. (2018). Landscape of pathogenic variations in a panel of 34 genes and cancer risk estimation from 5131 HBOC families. Genet. Med..

[70] Chapman J.R., Sossick A.J., Boulton S.J., Jackson S.P. (2012). BRCA1-associated exclusion of 53BP1 from DNA damage sites underlies temporal control of DNA repair. J. Cell Sci..

[71] Endl E., Gerdes J. (2000). The Ki-67 Protein: Fascinating Forms and an Unknown Function. Exp. Cell Res..

[72] Cuzick J., Dowsett M., Pineda S., Wale C., Salter J., Quinn E., Zabaglo L., Mallon E., Green A.R., Ellis I.O. (2011). Prognostic Value of a Combined Estrogen Receptor, Progesterone Receptor, Ki-67, and Human Epidermal Growth Factor Receptor 2 Immunohistochemical Score and Comparison With the Genomic Health Recurrence Score in Early Breast Cancer. JCO.

[73] Cuylen S., Blaukopf C., Politi A.Z., Müller-Reichert T., Neumann B., Poser I., Ellenberg J., Hyman A.A., Gerlich D.W. (2016). Ki-67 acts as a biological surfactant to disperse mitotic chromosomes. Nature.

[74] Sobecki M., Mrouj K., Camasses A., Parisis N., Nicolas E., Llères D., Gerbe F., Prieto S., Krasinska L., David A. (2016). The cell proliferation antigen Ki-67 organises heterochromatin. eLife.

[75] Takagi M., Natsume T., Kanemaki M.T., Imamoto N. (2016). Perichromosomal protein Ki67 supports mitotic chromosome architecture. Genes Cells.

[76] Sobecki M., Mrouj K., Colinge J., Gerbe F., Jay P., Krasinska L., Dulic V., Fisher D. (2017). Cell-Cycle Regulation Accounts for Variability in Ki-67 Expression Levels. Cancer Res..

[77] Miller I., Min M., Yang C., Tian C., Gookin S., Carter D., Spencer S.L. (2018). Ki67 is a Graded Rather than a Binary Marker of Proliferation versus Quiescence. Cell Rep..

[78] Dexter D.L., Kowalski H.M., Blazar B.A., Fligiel Z., Vogel R., Heppner G.H. (1978). Heterogeneity of tumor cells from a single mouse mammary tumor. Cancer Res..

[79] Mrouj K., Singh P., Sobecki M., Dubra G., Al Ghoul E., Aznar A., Prieto S., Pirot N., Bernex F., Bordignon B. (2019). Ki-67 promotes sequential stages of tumourigenesis by enabling cellular plasticity. Cancer Biol..

[80] Redig A.J., McAllister S.S. (2013). Breast cancer as a systemic disease: A view of metastasis. J. Intern. Med..

[81] Chambers A.F., Groom A.C., MacDonald I.C. (2002). Dissemination and growth of cancer cells in metastatic sites. Nat. Rev. Cancer.

[82] Ocaña O.H., Córcoles R., Fabra Á., Moreno-Bueno G., Acloque H., Vega S., Barrallo-Gimeno A., Cano A., Nieto M.A. (2012). Metastatic Colonization Requires the Repression of the Epithelial-Mesenchymal Transition Inducer Prrx1. Cancer Cell.

[83] Maitra A. (2019). Molecular envoys pave the way for pancreatic cancer to invade the liver. Nature.

[84] Massagué J., Obenauf A.C. (2016). Metastatic colonization by circulating tumour cells. Nature.

[85] Kalluri R., Neilson E.G. (2003). Epithelial-mesenchymal transition and its implications for fibrosis. J. Clin. Investig..

[86] Nieto M.A. (2011). The Ins and Outs of the Epithelial to Mesenchymal Transition in Health and Disease. Annu. Rev. Cell Dev. Biol..

[87] Wellner U., Schubert J., Burk U.C., Schmalhofer O., Zhu F., Sonntag A., Waldvogel B., Vannier C., Darling D., zur Hausen A. (2009). The EMT-activator ZEB1 promotes tumorigenicity by repressing stemness-inhibiting microRNAs. Nat. Cell Biol..

[88] Kushwaha P.P., Gupta S., Singh A.K., Kumar S. (2019). Emerging Role of Migration and Invasion Enhancer 1 (MIEN1) in Cancer Progression and Metastasis. Front. Oncol..

[89] Kpetemey M., Dasgupta S., Rajendiran S., Das S., Gibbs L.D., Shetty P., Gryczynski Z., Vishwanatha J.K. (2015). MIEN1, a novel interactor of Annexin A2, promotes tumor cell migration by enhancing AnxA2 cell surface expression. Mol. Cancer.

[90] Dasgupta S., Wasson L.M., Rauniyar N., Prokai L., Borejdo J., Vishwanatha J.K. (2009). Novel gene C17orf37 in 17q12 amplicon promotes migration and invasion of prostate cancer cells. Oncogene.

[91] Dasgupta S., Cushman I., Kpetemey M., Casey P.J., Vishwanatha J.K. (2011). Prenylated C17orf37 Induces Filopodia Formation to Promote Cell Migration and Metastasis. J. Biol. Chem..

[92] Van Treuren T., Vishwanatha J.K. (2018). CRISPR deletion of MIEN1 in breast cancer cells. PLoS ONE.

[93] Shen F., Zhang Y., Jernigan D.L., Feng X., Yan J., Garcia F.U., Meucci O., Salvino J.M., Fatatis A. (2016). Novel Small-Molecule CX3CR1 Antagonist Impairs Metastatic Seeding and Colonization of Breast Cancer Cells. Mol. Cancer Res..

[94] Bazan J.F., Bacon K.B., Hardiman G., Wang W., Soo K., Rossi D., Greaves D.R., Zlotnik A., Schall T.J. (1997). A new class of membrane-bound chemokine with a CX3C motif. Nature.

[95] Onitilo A.A., Engel J.M., Greenlee R.T., Mukesh B.N. (2009). Breast Cancer Subtypes Based on ER/PR and Her2 Expression: Comparison of Clinicopathologic Features and Survival. Clin. Med. Res..

[96] Gruber I., Fehm T., Taran F.A., Wallwiener M., Hahn M., Wallwiener D., Krawzyck N., Hoffmann J., Hartkopf A.D. (2014). Disseminated tumor cells as a monitoring tool for adjuvant therapy in patients with primary breast cancer. Breast Cancer Res. Treat..

[97] Cook D.N., Chen S.-C., Sullivan L.M., Manfra D.J., Wiekowski M.T., Prosser D.M., Vassileva G., Lira S.A. (2001). Generation and Analysis of Mice Lacking the Chemokine Fractalkine. Mol. Cell. Biol..

[98] Jamieson-Gladney W.L., Zhang Y., Fong A.M., Meucci O., Fatatis A. (2011). The chemokine receptor CX3CR1 is directly involved in the arrest of breast cancer cells to the skeleton. Breast Cancer Res..

[99] Xie K. (2001). Interleukin-8 and human cancer biology. Cytokine Growth Factor Rev..

[100] Long X., Ye Y., Zhang L., Liu P., Yu W., Wei F., Ren X., Yu J. (2016). IL-8, a novel messenger to cross-link inflammation and tumor EMT via autocrine and paracrine pathways (Review). Int. J. Oncol..

[101] Stillie R., Farooq S.M., Gordon J.R., Stadnyk A.W. (2009). The functional significance behind expressing two IL-8 receptor types on PMN. J. Leukoc. Biol..

[102] Wuyts A., Van Osselaer N., Haelens A., Samson I., Herdewijn P., Ben-Baruch A., Oppenheim J.J., Proost P., Van Damme J. (1997). Characterization of Synthetic Human Granulocyte Chemotactic Protein 2: Usage of Chemokine Receptors CXCR1 and CXCR2 and in Vivo Inflammatory Properties. Biochemistry.

[103] Saintigny P., Massarelli E., Lin S., Ahn Y.-H., Chen Y., Goswami S., Erez B., O’Reilly M.S., Liu D., Lee J.J. (2013). CXCR2 Expression in Tumor Cells Is a Poor Prognostic Factor and Promotes Invasion and Metastasis in Lung Adenocarcinoma. Cancer Res..

[104] Jin K., Pandey N.B., Popel A.S. (2017). Crosstalk between stromal components and tumor cells of TNBC via secreted factors enhances tumor growth and metastasis. Oncotarget.

[105] Sun X., Cheng G., Hao M., Zheng J., Zhou X., Zhang J., Taichman R.S., Pienta K.J., Wang J. (2010). CXCL12/CXCR4/CXCR7 chemokine axis and cancer progression. Cancer Metastasis Rev..

[106] Yang M., Zeng C., Li P., Qian L., Ding B., Huang L., Li G., Jiang H., Gong N., Wu W. (2019). Impact of CXCR4 and CXCR7 knockout by CRISPR/Cas9 on the function of triple-negative breast cancer cells. OncoTargets Ther..

[107] Ganju R.K., Brubaker S.A., Meyer J., Dutt P., Yang Y., Qin S., Newman W., Groopman J.E. (1998). The α-Chemokine, Stromal Cell-derived Factor-1α, Binds to the Transmembrane G-protein-coupled CXCR-4 Receptor and Activates Multiple Signal Transduction Pathways. J. Biol. Chem..

[108] Miao Z., Luker K.E., Summers B.C., Berahovich R., Bhojani M.S., Rehemtulla A., Kleer C.G., Essner J.J., Nasevicius A., Luker G.D. (2007). CXCR7 (RDC1) promotes breast and lung tumor growth in vivo and is expressed on tumor-associated vasculature. Proc. Natl. Acad. Sci. USA.

[109] Wang J., Shiozawa Y., Wang J., Wang Y., Jung Y., Pienta K.J., Mehra R., Loberg R., Taichman R.S. (2008). The Role of CXCR7/RDC1 as a Chemokine Receptor for CXCL12/SDF-1 in Prostate Cancer. J. Biol. Chem..

[110] Zheng K., Li H.-Y., Su X.-L., Wang X.-Y., Tian T., Li F., Ren G.-S. (2010). Chemokine receptor CXCR7 regulates the invasion, angiogenesis and tumor growth of human hepatocellular carcinoma cells. J. Exp. Clin. Cancer Res..

[111] Burns J.M., Summers B.C., Wang Y., Melikian A., Berahovich R., Miao Z., Penfold M.E.T., Sunshine M.J., Littman D.R., Kuo C.J. (2006). A novel chemokine receptor for SDF-1 and I-TAC involved in cell survival, cell adhesion, and tumor development. J. Exp. Med..

[112] Wu W., Qian L., Dai J., Ding B., Chen X. (2017). Expression of chemokine CXCL12 and its receptor (CXCR4 and CXCR7) in different molecular subtypes of human breast carcinoma and the clinical significance. Zhong Nan Da Xue Xue Bao Yi Xue Ban.

[113] Johnson G.L. (2002). Mitogen-Activated Protein Kinase Pathways Mediated by ERK, JNK, and p38 Protein Kinases. Science.

[114] Rattanasinchai C., Gallo K. (2016). MLK3 Signaling in Cancer Invasion. Cancers.

[115] Cronan M.R., Nakamura K., Johnson N.L., Granger D.A., Cuevas B.D., Wang J.-G., Mackman N., Scott J.E., Dohlman H.G., Johnson G.L. (2012). Defining MAP3 kinases required for MDA-MB-231 cell tumor growth and metastasis. Oncogene.

[116] Jiang X., Xie H., Dou Y., Yuan J., Zeng D., Xiao S. (2020). Expression and function of FRA1 protein in tumors. Mol. Biol. Rep..

[117] Kessenbrock K., Plaks V., Werb Z. (2010). Matrix Metalloproteinases: Regulators of the Tumor Microenvironment. Cell.

[118] Rattanasinchai C., Llewellyn B.J., Conrad S.E., Gallo K.A. (2017). MLK3 regulates FRA-1 and MMPs to drive invasion and transendothelial migration in triple-negative breast cancer cells. Oncogenesis.

[119] Kohli S., Kohli V. (2011). Role of RANKL-RANK/osteoprotegerin molecular complex in bone remodeling and its immunopathologic implications. Indian J. Endocr. Metab..

[120] Cody J.J., Rivera A.A., Lyons G.R., Yang S.W., Wang M., Sarver D.B., Wang D., Selander K.S., Kuo H.-C., Meleth S. (2010). Arming a replicating adenovirus with osteoprotegerin reduces the tumor burden in a murine model of osteolytic bone metastases of breast cancer. Cancer Gene.

[121] Sheridan J.P. (1997). Control of TRAIL-Induced Apoptosis by a Family of Signaling and Decoy Receptors. Science.

[122] Geerts D., Chopra C., Connelly L. (2020). Osteoprotegerin: Relationship to Breast Cancer Risk and Prognosis. Front. Oncol..

[123] Zhong Z., Niu P., Wang M., Huang G., Xu S., Sun Y., Xu X., Hou Y., Sun X., Yan Y. (2016). Targeted disruption of sp7 and myostatin with CRISPR-Cas9 results in severe bone defects and more muscular cells in common carp. Sci. Rep..

[124] Kichev A., Eede P., Gressens P., Thornton C., Hagberg H. (2017). Implicating Receptor Activator of NF-κB (RANK)/RANK Ligand Signalling in Microglial Responses to Toll-Like Receptor Stimuli. Dev. Neurosci..

[125] Goswami S., Sharma-Walia N. (2016). Crosstalk between osteoprotegerin (OPG), fatty acid synthase (FASN) and, cycloxygenase-2 (COX-2) in breast cancer: Implications in carcinogenesis. Oncotarget.

[126] Song L., Luo Z.-Q. (2019). Post-translational regulation of ubiquitin signaling. J. Cell Biol..

[127] Shearer R.F., Iconomou M., Watts C.K.W., Saunders D.N. (2015). Functional Roles of the E3 Ubiquitin Ligase UBR5 in Cancer. Mol. Cancer Res..

[128] Liao L., Song M., Li X., Tang L., Zhang T., Zhang L., Pan Y., Chouchane L., Ma X. (2017). E3 Ubiquitin Ligase UBR5 Drives the Growth and Metastasis of Triple-Negative Breast Cancer. Cancer Res..

[129] Mavaddat N., Barrowdale D., Andrulis I.L., Domchek S.M., Eccles D., Nevanlinna H., Ramus S.J., Spurdle A., Robson M., Sherman M. (2012). Pathology of Breast and Ovarian Cancers among BRCA1 and BRCA2 Mutation Carriers: Results from the Consortium of Investigators of Modifiers of BRCA1/2 (CIMBA). Cancer Epidemiol. Biomark. Prev..

[130] Wilson J.R.F., Bateman A.C., Hanson H., An Q., Evans G., Rahman N., Jones J.L., Eccles D.M. (2010). A novel HER2-positive breast cancer phenotype arising from germline TP53 mutations. J. Med. Genet..

[131] Bouwman P., Aly A., Escandell J.M., Pieterse M., Bartkova J., van der Gulden H., Hiddingh S., Thanasoula M., Kulkarni A., Yang Q. (2010). 53BP1 loss rescues BRCA1 deficiency and is associated with triple-negative and BRCA-mutated breast cancers. Nat. Struct. Mol. Biol..

[132] Ahmed A., Ashraf D., Bahaa A., El-Tayebi H., Adwan H. (2020). Impact of CDK4 knock out using CRISPR/Cas9 gene editing technology on breast cancer progression. Eur. J. Cancer.

[133] Xu K., Chen G., Li X., Wu X., Chang Z., Xu J., Zhu Y., Yin P., Liang X., Dong L. (2017). MFN2 suppresses cancer progression through inhibition of mTORC2/Akt signaling. Sci. Rep..

[134] Mendes de Almeida R., Bandarra S., Clara Ribeiro A., Mascarenhas P., Bekman E., Barahona I. (2019). Inactivation of APOBEC3G gene in breast cancer cells using the CRISPR/Cas9 system. Ann. Med..

[135] Heidary Arash E., Shiban A., Song S., Attisano L. (2017). MARK4 inhibits Hippo signaling to promote proliferation and migration of breast cancer cells. EMBO Rep..

[136] Álvarez-Fernández M., Sanz-Flores M., Sanz-Castillo B., Salazar-Roa M., Partida D., Zapatero-Solana E., Ali H.R., Manchado E., Lowe S., VanArsdale T. (2017). Therapeutic relevance of the PP2A-B55 inhibitory kinase MASTL/Greatwall in breast cancer. Cell Death Differ..

[137] Lin A., Giuliano C.J., Sayles N.M., Sheltzer J.M. (2017). CRISPR/Cas9 mutagenesis invalidates a putative cancer dependency targeted in on-going clinical trials. eLife.

[138] Tian J., Wang V., Wang N., Khadang B., Boudreault J., Bakdounes K., Ali S., Lebrun J.-J. (2021). Identification of MFGE8 and KLK5/7 as mediators of breast tumorigenesis and resistance to COX-2 inhibition. Breast Cancer Res..

[139] Kim D., Bae S., Park J., Kim E., Kim S., Yu H.R., Hwang J., Kim J.-I., Kim J.-S. (2015). Digenome-seq: Genome-wide profiling of CRISPR-Cas9 off-target effects in human cells. Nat. Methods.

[140] Wilbie D., Walther J., Mastrobattista E. (2019). Delivery Aspects of CRISPR/Cas for in Vivo Genome Editing. Acc. Chem. Res..

[141] Kouranova E., Forbes K., Zhao G., Warren J., Bartels A., Wu Y., Cui X. (2016). CRISPRs for Optimal Targeting: Delivery of CRISPR Components as DNA, RNA, and Protein into Cultured Cells and Single-Cell Embryos. Hum. Gene Ther..

[142] Luther D.C., Lee Y.W., Nagaraj H., Scaletti F., Rotello V.M. (2018). Delivery approaches for CRISPR/Cas9 therapeutics in vivo: Advances and challenges. Expert Opin. Drug Deliv..

[143] Gray S.J., Woodard K.T., Samulski R.J. (2010). Viral vectors and delivery strategies for CNS gene therapy. Ther. Deliv..

[144] Chandrasekaran A.P., Song M., Kim K.-S., Ramakrishna S. (2018). Different Methods of Delivering CRISPR/Cas9 Into Cells. Progress in Molecular Biology and Translational Science.

[145] Rauschhuber C., Noske N., Ehrhardt A. (2012). New insights into stability of recombinant adenovirus vector genomes in mammalian cells. Eur. J. Cell Biol..

[146] Crystal R.G. (2014). Adenovirus: The First Effective In Vivo Gene Delivery Vector. Hum. Gene Ther..

[147] Lee C.S., Bishop E.S., Zhang R., Yu X., Farina E.M., Yan S., Zhao C., Zeng Z., Shu Y., Wu X. (2017). Adenovirus-Mediated Gene Delivery: Potential Applications for Gene and Cell-Based Therapies in the New Era of Personalized Medicine. Genes Dis..

[148] Xu C.L., Ruan M.Z.C., Mahajan V.B., Tsang S.H. (2019). Viral Delivery Systems for CRISPR. Viruses.

[149] Ahi Y.S., Bangari D.S., Mittal S.K. (2011). Adenoviral Vector Immunity: Its Implications and Circumvention Strategies. Curr. Gene Ther..

[150] Ricobaraza A., Gonzalez-Aparicio M., Mora-Jimenez L., Lumbreras S., Hernandez-Alcoceba R. (2020). High-Capacity Adenoviral Vectors: Expanding the Scope of Gene Therapy. Int. J. Mol. Sci..

[151] Liu C., Zhang L., Liu H., Cheng K. (2017). Delivery strategies of the CRISPR-Cas9 gene-editing system for therapeutic applications. J. Control Release.

[152] Wanisch K., Yáñez-Muñoz R.J. (2009). Integration-deficient Lentiviral Vectors: A Slow Coming of Age. Mol. Ther..

[153] Mingozzi F., High K.A. (2011). Therapeutic in vivo gene transfer for genetic disease using AAV: Progress and challenges. Nat. Rev. Genet..

[154] Lau C.-H., Suh Y. (2017). In vivo genome editing in animals using AAV-CRISPR system: Applications to translational research of human disease. F1000Research.

[155] Linden R.M., Ward P., Giraud C., Winocour E., Berns K.I. (1996). Site-specific integration by adeno-associated virus. Proc. Natl. Acad. Sci. USA.

[156] Nelson C.E., Gersbach C.A. (2016). Engineering Delivery Vehicles for Genome Editing. Annu. Rev. Chem. Biomol. Eng..

[157] Hermonat P.L., Quirk J.G., Bishop B.M., Han L. (1997). The packaging capacity of adeno-associated virus (AAV) and the potential for wild-type-plus AAV gene therapy vectors. FEBS Lett..

[158] Ramamoorth M. (2015). Non Viral Vectors in Gene Therapy—An Overview. J. Clin. Diagn. Res..

[159] Young J.L., Dean D.A. (2015). Electroporation-Mediated Gene Delivery. Advances in Genetics.

[160] Rubinsky B. (2007). Irreversible Electroporation in Medicine. Technol Cancer Res. Treat..

[161] Dean D.A., Gasiorowski J.Z. (2011). Microinjecting Cells Using a Constant-Flow Microinjection System. Cold Spring Harb. Protoc..

[162] Luong L.N., McFalls K.M., Kohn D.H. (2009). Gene delivery via DNA incorporation within a biomimetic apatite coating. Biomaterials.

[163] Chowdhury E.H., Sasagawa T., Nagaoka M., Kundu A.K., Akaike T. (2003). Transfecting mammalian cells by DNA/calcium phosphate precipitates: Effect of temperature and pH on precipitation. Anal. Biochem..

[164] Daraee H., Etemadi A., Kouhi M., Alimirzalu S., Akbarzadeh A. (2016). Application of liposomes in medicine and drug delivery. Artif. Cells Nanomed. Biotechnol..

[165] Bozzuto G., Molinari A. (2015). Liposomes as nanomedical devices. Int. J. Nanomed..

[166] Shalaby K., Aouida M., El-Agnaf O. (2020). Tissue-Specific Delivery of CRISPR Therapeutics: Strategies and Mechanisms of Non-Viral Vectors. Int. J. Mol. Sci..

[167] Cheng Q., Wei T., Farbiak L., Johnson L.T., Dilliard S.A., Siegwart D.J. (2020). Selective organ targeting (SORT) nanoparticles for tissue-specific mRNA delivery and CRISPR—Cas gene editing. Nat. Nanotechnol..

[168] Chen Z., Liu F., Chen Y., Liu J., Wang X., Chen A.T., Deng G., Zhang H., Liu J., Hong Z. (2017). Targeted Delivery of CRISPR/Cas9-Mediated Cancer Gene Therapy via Liposome-Templated Hydrogel Nanoparticles. Adv. Funct. Mater..

[169] Wei T., Cheng Q., Min Y.-L., Olson E.N., Siegwart D.J. (2020). Systemic nanoparticle delivery of CRISPR-Cas9 ribonucleoproteins for effective tissue specific genome editing. Nat. Commun..

[170] Maffei M., Morelli C., Graham E., Patriarca S., Donzelli L., Doleschall B., de Castro Reis F., Nocchi L., Chadick C.H., Reymond L. (2019). A ligand-based system for receptor-specific delivery of proteins. Sci. Rep..

[171] Zhuang J., Tan J., Wu C., Zhang J., Liu T., Fan C., Li J., Zhang Y. (2020). Extracellular vesicles engineered with valency-controlled DNA nanostructures deliver CRISPR/Cas9 system for gene therapy. Nucleic Acids Res..

[172] Frangoul H., Altshuler D., Cappellini M.D., Chen Y.-S., Domm J., Eustace B.K., Foell J., de la Fuente J., Grupp S., Handgretinger R. (2021). CRISPR-Cas9 Gene Editing for Sickle Cell Disease and β-Thalassemia. N. Engl. J. Med..

[173] Ding Q., Regan S.N., Xia Y., Oostrom L.A., Cowan C.A., Musunuru K. (2013). Enhanced Efficiency of Human Pluripotent Stem Cell Genome Editing through Replacing TALENs with CRISPRs. Cell Stem Cell.

[174] Li S., Garay J.P., Tubbs C.A., Franco H.L. (2021). CRISPR-based knock-in mutagenesis of the pioneer transcription factor FOXA1: Optimization of strategies for multi-allelic proteins in cancer cells. FEBS Open Bio..

[175] Wang W., Malyutina A., Pessia A., Saarela J., Heckman C.A., Tang J. (2019). Combined gene essentiality scoring improves the prediction of cancer dependency maps. EBioMedicine.

[176] Chen A., Wen S., Liu F., Zhang Z., Liu M., Wu Y., He B., Yan M., Kang T., Lam E.W. (2021). CRISPR/Cas9 screening identifies a kinetochore-microtubule dependent mechanism for Aurora-A inhibitor resistance in breast cancer. Cancer Commun..

[177] Ran F.A., Hsu P.D., Lin C.-Y., Gootenberg J.S., Konermann S., Trevino A.E., Scott D.A., Inoue A., Matoba S., Zhang Y. (2013). Double Nicking by RNA-Guided CRISPR Cas9 for Enhanced Genome Editing Specificity. Cell.

[178] Kleinstiver B.P., Pattanayak V., Prew M.S., Tsai S.Q., Nguyen N.T., Zheng Z., Joung J.K. (2016). High-fidelity CRISPR–Cas9 nucleases with no detectable genome-wide off-target effects. Nature.

[179] Hiranniramol K., Chen Y., Liu W., Wang X. (2020). Generalizable sgRNA design for improved CRISPR/Cas9 editing efficiency. Bioinformatics.

[180] Walton R.T., Christie K.A., Whittaker M.N., Kleinstiver B.P. (2020). Unconstrained genome targeting with near-PAMless engineered CRISPR-Cas9 variants. Science.

[181] Kim S., Kim D., Cho S.W., Kim J., Kim J.-S. (2014). Highly efficient RNA-guided genome editing in human cells via delivery of purified Cas9 ribonucleoproteins. Genome Res..

[182] Baylis F., McLeod M. (2018). First-in-human Phase 1 CRISPR Gene Editing Cancer Trials: Are We Ready?. Curr. Gene Ther..

[183] Yu K.-R., Natanson H., Dunbar C.E. (2016). Gene Editing of Human Hematopoietic Stem and Progenitor Cells: Promise and Potential Hurdles. Hum. Gene Ther..

[184] Tong S., Moyo B., Lee C.M., Leong K., Bao G. (2019). Engineered materials for in vivo delivery of genome-editing machinery. Nat. Rev. Mater..

[185] Uddin F., Rudin C.M., Sen T. (2020). CRISPR Gene Therapy: Applications, Limitations, and Implications for the Future. Front. Oncol..

[186] Cyranoski D. (2019). The CRISPR-baby scandal: What’s next for human gene-editing. Nature.

[187] Brokowski C., Adli M. (2019). CRISPR Ethics: Moral Considerations for Applications of a Powerful Tool. J. Mol. Biol..

[188] Andorno R., Baylis F., Darnovsky M., Dickenson D., Haker H., Hasson K., Lowthorp L., Annas G.J., Bourgain C., Drabiak K. (2020). Geneva Statement on Heritable Human Genome Editing: The Need for Course Correction. Trends Biotechnol..

